# Marital status and incident screen-positive cognitive impairment in a Taiwanese longitudinal cohort (2005–2016): Associations vary by cognitive screening instrument

**DOI:** 10.1371/journal.pone.0359778

**Published:** 2026-10-05

**Authors:** Yi-Chao Zhou, Yi-Chang Chou, Yin-Ling Hung, Yen-Jung Chang

**Affiliations:** 1 Department of Health Promotion and Health Education, College of Education, National Taiwan Normal University, Taipei, Taiwan; 2 Department of Family and Community Medicine, Tri-Service General Hospital, National Defense Medical University, Taipei, Taiwan; 3 School of Medicine, National Defense Medical University, Taipei, Taiwan; 4 Department of Family Medicine, Taichung Armed Forces General Hospital, Taichung, Taiwan; 5 Department of Education and Research, Taipei City Hospital, Taipei, Taiwan; 6 University of Taipei, Taipei, Taiwan; 7 Department of Nursing, Tri-Service General Hospital, National Defense Medical University, Taipei, Taiwan; NED University of Engineering and Technology, PAKISTAN

## Abstract

Marital status has been associated with cognitive aging; however, whether such associations vary according to cognitive assessment modality remains unclear. This study examined whether associations between marital status and incident screen-positive cognitive impairment (MCI) differ by screening instrument and population subgroups in a large longitudinal cohort of community-dwelling older adults in Taiwan. We analyzed 103,863 adults aged ≥65 years from the Taipei City Elderly Health Examination Database (2005–2016). Cognitive status was assessed using the Short Portable Mental Status Questionnaire (SPMSQ; 2005–2011) and the 8-item Ascertain Dementia questionnaire (AD8; 2012–2016). Baseline marital status was categorized as married, divorced, widowed, or never married. Cox proportional hazards models with time-updated covariates were fitted separately for each assessment period, adjusting for demographic, socioeconomic, health, and lifestyle factors. Sensitivity analyses were conducted to examine potential reverse causation and variation in follow-up duration. Among 61,304 participants assessed during the SPMSQ period, never-married individuals had a higher risk of incident MCI compared with married individuals (hazard ratio [HR] 1.37, 95% confidence interval [CI] 1.08–1.74). In contrast, among 42,559 participants assessed during the AD8 period, never-married status was associated with a lower risk of MCI (HR 0.60, 95% CI 0.46–0.77). Effect modification by sex and age was observed across periods. Sensitivity analyses yielded consistent findings. Associations between marital status and incident MCI differed across assessment periods that varied in screening instrument, follow-up duration, and population composition; this measurement heterogeneity, rather than a confirmed temporal change, may account for the reversal observed among never-married participants. These findings suggest that interpretation of marital status–cognition associations should be considered alongside the measurement approach and study period under which they were derived.

## Introduction

The rising prevalence of dementia among aging populations represents a critical global health challenge, with cases projected to triple to 152 million by 2050 [1–3], significantly impacting quality of life and healthcare sustainability [4,5]. Understanding modifiable risk factors for cognitive decline has become increasingly urgent given demographic shifts toward rapidly aging populations, particularly in societies experiencing compressed aging trajectories [6]. Marital status has emerged as a potentially important protective factor against cognitive decline, with extensive research examining how marital relationships affect brain health and cognitive resilience [7,8]. Theoretically, the continuous mental stimulation from daily marital interactions may contribute to enhanced cognitive reserve through sustained social engagement, emotional support, and shared cognitive activities [9]. Although mild marital stress may benefit cognitive function through adaptive challenge [10], predominantly negative marital experiences are associated with increased cognitive decline risk [11]. Nevertheless, findings regarding the protective effects of marriage remain inconsistent [12,13]. Large epidemiological studies have identified higher rates of cognitive impairment among never-married individuals [14], yet other studies suggest that unmarried older adults may actually have a lower risk of dementia after adjusting for socioeconomic and health factors [12]. The role of biological sex further complicates these associations. Some studies indicate that marital dissolution through divorce or spousal death disproportionately increases cognitive vulnerability in men, possibly reflecting differences in social network maintenance and emotional coping strategies [13,15]. However, other studies have found no significant sex differences after adjusting for socioeconomic and other confounding factors [16].

A fundamental but underappreciated source of inconsistency in this literature is how cognitive impairment is operationalized and measured. Performance-based tests (e.g., Short Portable Mental Status Questionnaire, SPMSQ) directly assess cognitive abilities through structured tasks, while informant-based questionnaires (e.g., 8-item Ascertain Dementia, AD8) rely on reports of functional decline [17,18]. These modalities capture distinct aspects: performance-based tests measure current cognitive capacity, whereas informant-based tools detect changes from prior baseline and may better reflect functional impact. Critically, informant-based assessments may be susceptible to differential reporting bias—married individuals typically have co-residing spouses who detect subtle changes, whereas never-married individuals may rely on self-report or less frequent observers. Despite this, few studies have systematically compared marital status-cognition associations across assessment modalities within the same population.

A related conceptual challenge in this literature is the possibility of reverse causality [15,19]. Rather than marital status causally shaping cognitive trajectories, incipient cognitive decline—even at a subclinical stage, before it would be detected on a screening instrument—may itself influence an individual’s likelihood of marrying, remaining married, or experiencing marital dissolution. Individuals with early, undetected cognitive changes may be less likely to initiate or sustain a long-term marriage, more likely to experience divorce or separation as a result of relationship strain, or, conversely, more likely to remain married if a spouse compensates for emerging functional difficulties in ways that delay formal detection. This possibility is particularly salient in observational cohorts with infrequent assessment intervals, where subclinical decline may precede the first recorded cognitive assessment by several years. Although our analytic strategy includes lag analyses intended to mitigate this concern (see Methods), the baseline ascertainment of marital status cannot fully rule out reverse causal pathways, and this conceptual limitation should be borne in mind when interpreting the associations reported below.

This study addresses this gap using a large longitudinal cohort of community-dwelling older adults in Taiwan, spanning a transition between two screening instruments (SPMSQ 2005–2011 versus AD8 2012–2016). Taiwan’s rapid demographic transition (projected super-aged society by 2025) and universal healthcare system provide a unique context for examining whether marital status-cognition associations differ across measurement approaches and are modified by sex, age, education, and health status. We hypothesized meaningful heterogeneity across both measurement approaches and population strata. Findings will clarify how measurement choices influence observed associations, identify vulnerable subgroups, and inform the development of targeted cognitive health interventions for diverse older populations in rapidly aging societies.

## Materials and methods

### Study population

This 11-year longitudinal prospective cohort study utilized data from the Taipei City Elderly Health Examination Database, established through collaboration between Taipei City Hospital and the Taipei City Department of Health. The database aggregates annual health examination data from multiple public and private medical institutions. The dataset covering the study period (2005–2016) was accessed for research purposes on 01/06/2025. The study population comprised adults aged ≥65 years participating in government-sponsored annual health examinations (2005‒2016), including standardized physical/oral examinations and self-administered questionnaires. Of the 184,403 individuals identified, participants were screened per predefined eligibility criteria (Fig 1). Exclusion criteria included: single examination records (n = 44,254 for Short Portable Mental Status Questionnaire [SPMSQ]; n = 32,700 for 8-item Ascertain Dementia [AD8]), baseline dementia medication use (n = 399 for SPMSQ; n = 411 for AD8), and incomplete questionnaire data (n = 1,185 for SPMSQ; n = 709 for AD8). The final analysis included 103,863 participants (n = 61,304 for SPMSQ; n = 42,559 for AD8) with mean follow-up periods of 3.7 years (SPMSQ) and 2.8 years (AD8). The study was conducted in accordance with the principles of the 1964 Helsinki Declaration. The data were anonymized and encrypted prior to analysis, and the Institutional Review Board of Taipei City Hospital approved the study and granted an exemption from the requirement for written informed consent given that the data were originally collected for public health surveillance and research purposes (IRB No. TCHIRB-11303014-E).

**Fig 1:**
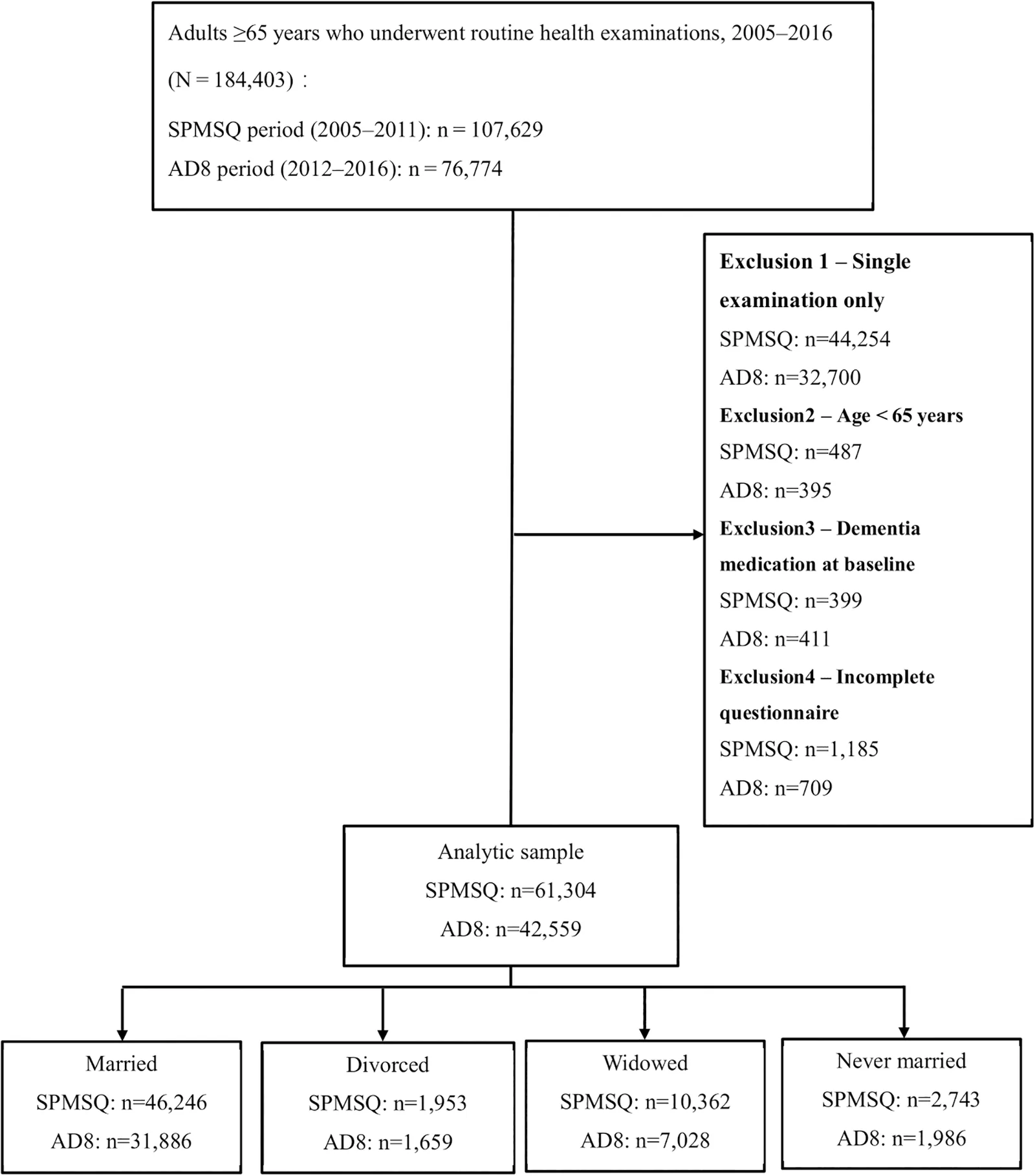
Flow chart of the patient selection process.

### Measures of cognitive function

In this study, screen-positive cognitive impairment (hereafter referred to as MCI for brevity and consistency with prior literature using these instruments) was ascertained using the SPMSQ and AD8 questionnaires. As both the SPMSQ and AD8 are validated screening tools rather than clinical diagnostic instruments, the resulting classification reflects screen-positive status based on a standardized cutoff rather than a clinical diagnosis of mild cognitive impairment or dementia. The SPMSQ serves as an established cognitive assessment instrument designed for older adults [20]. It comprises ten questions that evaluate multiple cognitive domains, encompassing temporal and spatial orientation, recall abilities, general knowledge awareness, and arithmetic skills. Scoring methodology is based on the number of incorrect responses, where each wrong answer receives a score of “1” while correct answers receive a score of “0.” The resulting assessment ranges from 0–10 points. Scores between 0‒2 suggest preserved cognitive function, whereas scores ≥3 indicate MCI. The AD8 functions as a cognitive decline detection instrument comprising eight evaluation criteria [21]. Each criterion utilizes a binary scoring system: 0 represents “no observable change,” while 1 indicates “change detected.” The assessment targets functional cognitive abilities across various life domains: decision-making capacity, engagement levels, repetitive behaviors, acquisition of new information, spatial-temporal awareness, monetary management, scheduling adherence, and cognitive coherence. A cumulative AD8 score of ≥2 indicates MCI. This assessment tool offers flexibility in administration, allowing completion through either caregiver observation or individual self-evaluation; however, the database did not record which respondent type (self vs. caregiver/informant) was used for each assessment, precluding direct stratification by informant type. Both the SPMSQ and AD8 used in this study were traditional Chinese versions that have been well validated in the Taiwanese population.

### Measures of marital status

We measured marital status at baseline using a self-reported questionnaire. This variable was treated as a fixed characteristic throughout the follow-up period and included four categories: married (reference), divorced, widowed, and never-married.

### Covariates

Covariates were defined based on established epidemiological criteria [22,23]. They were categorized as either fixed baseline variables or time-dependent variables. Baseline covariates, assessed at the start of the study, included age, sex (male or female), educational attainment (<6, 7–9, or ≥10 years), low-income household status, and living arrangement (alone or with others). In Taiwan, low-income household status was defined as an average monthly disposable income per capita below 60% of the minimum subsistence level (<NT$19,013), verified annually through household registration records [22]. Time-dependent covariates were updated at each annual follow-up and included health-related variables, such as body mass index (BMI), categorized as underweight (<18.5 kg/m^2^), normal (18.5–24.0 kg/m^2^), overweight (24–27 kg/m^2^), and obese (≥27 kg/m^2^) [24]; chronic-disease conditions; psychiatric history; psychological distress; and health behaviors (current smoking and occasional or frequent drinking; both recorded as yes/no). The chronic-disease covariates—hypertension, diabetes, hyperlipidemia, heart disease, and stroke—were ascertained from the self-administered questionnaire on the basis of self-reported current medication use and its reported indication, with each condition coded as present (yes/no) when a participant reported taking medication for that condition (for stroke, an indication of stroke or cerebrovascular disease). Psychiatric history was defined in the same manner, as self-reported use of medication for a psychiatric or psychological indication (depression, anxiety, or other psychiatric or psychosomatic conditions) or of sedative-hypnotic medication for insomnia. Psychological distress was measured using the 5-item Brief Symptom Rating Scale (BSRS-5), a validated screening instrument included in the examination questionnaire, with the total score (range 0–20) dichotomized at the established cutoff of ≥6 to indicate the presence of psychological distress. Thus, the fully adjusted model (Model 2) included age, sex, education, low-income household status, living arrangement, BMI category, hypertension, diabetes, hyperlipidemia, heart disease, stroke, psychiatric history, psychological distress, current smoking, and alcohol consumption. None of these covariates were derived from clinical diagnoses or ICD-coded records: demographic, socioeconomic, health, and behavioral data were collected through self-administered questionnaires, BMI was calculated from physical examination measurements, and low-income status was determined through administrative data linkage.

### Statistical analyses

Baseline characteristics were compared across marital status groups using chi-square tests for categorical variables and one-way ANOVA for continuous variables.

We employed Cox proportional hazards regression models with time-dependent covariates to estimate hazard ratios (HRs) and 95% confidence intervals (CIs) for the association between baseline marital status and incident MCI, with “married” as the reference group. Analyses were conducted separately for the SPMSQ period (2005–2011) and AD8 period (2012–2016). Model 2 adjusted for baseline covariates (age, sex, education, living arrangement, low-income status) and time-dependent covariates reassessed at each follow-up (BMI, histories of hypertension, diabetes, dyslipidemia, heart disease, stroke, psychiatric history, psychological distress, smoking, alcohol consumption).

Participants who did not screen positive were censored at their last attended examination, with death treated as censoring. Because linked mortality data were available only through 2012, a competing-risks analysis was not performed, and hazard ratios are reported as cause-specific hazards. Analyses were restricted to participants with complete data on all model covariates (complete-case analysis); the numbers excluded for incomplete questionnaire data are shown in Fig 1.

Subgroup analyses explored potential effect modification. We stratified analyses by sex, age group (65–79 vs. ≥ 80 years), and education level (<10 vs. ≥ 10 years). To investigate factors modifying risk among never-married participants, we performed stratified analyses with interaction terms (Fig 2), examining sex, age, education, medicated chronic diseases, BMI, living arrangement, and psychological distress.

**Fig 2:**
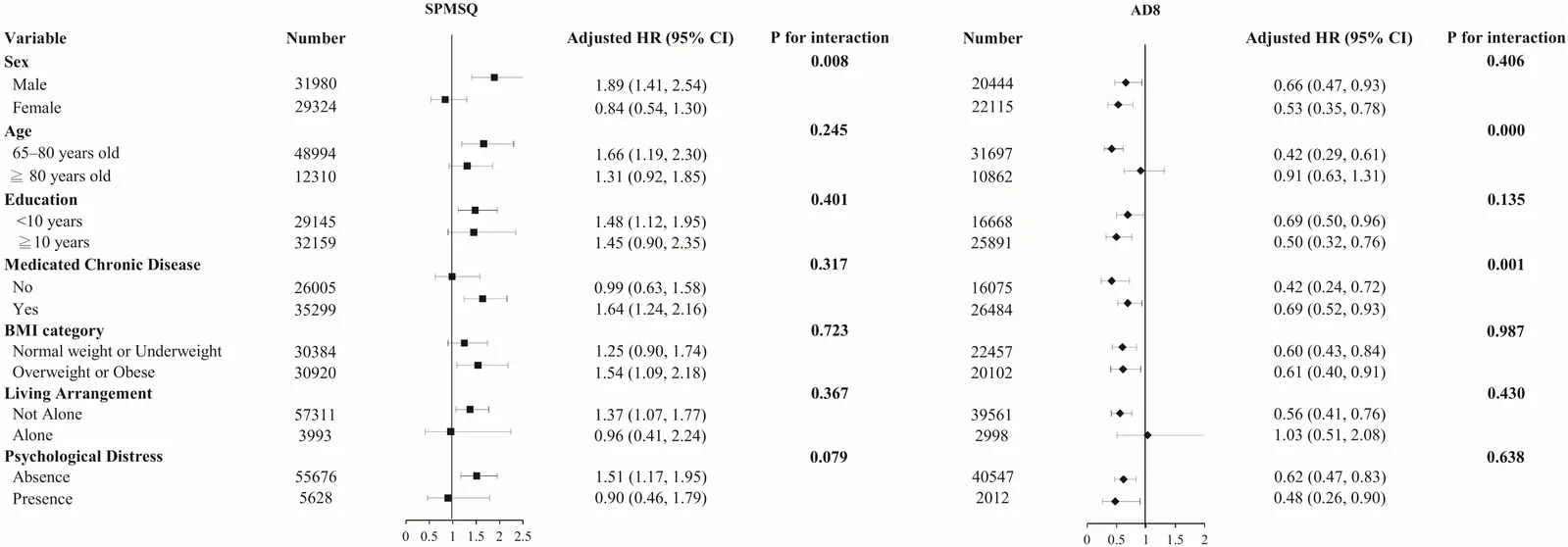
Subgroup analysis of the association between never-married status and incident screen-positive cognitive impairment. Because effect modification was examined across multiple subgroups, P-values for all marital status × modifier interactions were adjusted for multiple comparisons using the Benjamini–Hochberg false discovery rate across the full set of 14 interaction tests (S3 Table).Sensitivity analyses assessed robustness: (1) stratification by median follow-up duration (<4 vs. ≥ 4 years for SPMSQ; < 3 vs. ≥ 3 years for AD8); (2) exclusion of screen-positive MCI cases identified within the first 2 years to mitigate reverse causality. The proportional hazards assumption was evaluated by adding an interaction between marital status and log time to the fully adjusted model; because the counting-process formulation precludes the cumulative-residual supremum test, this time-interaction approach was used (S2 Table). As a further robustness check, marital status was re-specified as a time-varying covariate updated at each examination within the counting-process framework in the AD8 period; the estimates were materially unchanged (S1 Table).

All analyses used SAS 9.4 (SAS Institute, Cary, NC). Two-tailed P < 0.05 indicated statistical significance.

## Results

This study analyzed 61,304 (2005–2011) and 42,559 (2012–2016) participants, with married individuals comprising ~75% in both periods (Tables 1 and 2). Widowed participants were oldest (75.9–77.9 years), predominantly female (76.6–79.9%), and had highest hypertension (44.3–51.1%) and heart disease (20.1–22.7%) prevalence, but lowest smoking (2.8–4.7%) and alcohol consumption (10.3–11.6%). Never-married participants showed highest low-income rates (16.0% → 7.4%) and smoking prevalence (12.5% → 8.1%). Divorced individuals had highest rates of living alone (~31%), psychological distress (12.2% → 6.0%), and psychotropic medication use. Married participants were younger (73.5–74.5 years), had lowest low-income rates (1.5–3.5%), and highest alcohol consumption (~21–22%). These patterns remained consistent across both periods.

**Table 1:** Baseline characteristics and demographics of the study population in SPMSQ period (2005–2011) (n = 61,304).

Variables, n (%)	Married	Divorced	Widowed	Never married	*P* value
	46,246 (75.4)	1,953 (3.2)	10,362 (16.9)	2,743 (4.5)	
Age, years (mean±SD)	73.5 ± 6.0	73.7 ± 6.2	75.9 ± 6.4	75.4 ± 6.8	<.001^†^
Sex					<.001
Male	26,808 (58.0)	1,134 (58.1)	2,429 (23.4)	1,609 (58.7)	
Female	19,438 (42.0)	819 (41.9)	7,933 (76.6)	1,134 (41.3)	
Educational attainment (years)					<.001
≦6	13,779 (29.8)	572 (29.3)	4,704 (45.4)	1,130 (41.2)	
7–9	6,590 (14.2)	290 (14.9)	1,653 (16.0)	427 (15.6)	
≧10	25,877 (56.0)	1,091 (55.9)	4,005 (38.6)	1,186 (43.2)	
Low–income household	1,620 (3.5)	192 (9.8)	634 (6.1)	439 (16.0)	<.001
Living alone	641 (1.4)	602 (30.8)	2,284 (22.0)	466 (17.0)	<.001
BMI category					<.001
Underweight	1,521 (3.3)	81 (4.2)	423 (4.1)	159 (5.8)	
Normal weight	21,246 (45.9)	942 (48.2)	4,724 (45.6)	1,288 (47.0)	
Overweight	15,044 (32.5)	612 (31.3)	3,217 (31.1)	784 (28.6)	
Obesity	8,435 (18.2)	318 (16.3)	1,998 (19.3)	512 (18.7)	
Medication history					
Hypertension	18,727 (40.5)	747 (38.3)	4,590 (44.3)	1,122 (40.9)	<.001
Diabetes	5,489 (11.9)	249 (12.8)	1,290 (12.5)	347 (12.7)	0.187
Hyperlipidemia	3,329 (7.2)	128 (6.6)	801 (7.7)	142 (5.2)	<.001
Heart disease	8,191 (17.7)	315 (16.1)	2,084 (20.1)	507 (18.5)	<.001
Psychiatric history	1,555 (3.4)	87 (4.5)	456 (4.4)	99 (3.6)	<.001
Psychological distress	3,981 (8.6)	238 (12.2)	1,150 (11.1)	259 (9.4)	<.001
Current smoker	3,454 (7.5)	218 (11.2)	488 (4.7)	342 (12.5)	<.001
Occasional or frequent drinking	10,173 (22.0)	423 (21.7)	1,206 (11.6)	426 (15.5)	<.001

Note. SPMSQ, Short Portable Mental Status Questionnaire; AD8, Ascertain Dementia 8-item Informant Questionnaire; BMI, body mass index; married, married living together, and cohabiting; divorced, divorced and married living separately. The chi-square test was used for all variables, except for age, where ANOVA was used (indicated by †).

**Table 2:** Baseline characteristics and demographics of the study population in AD8 period (2012–2016) (n = 42,559).

Variables, n (%)	Married	Divorced	Widowed	Never married	*P* value
	31,886 (74.9)	1,659 (3.9)	7,028 (16.5)	1,986 (4.7)	
Age, years (mean±SD)	74.5 ± 6.5	74.0 ± 7.0	77.9 ± 6.8	73.5 ± 7.2	<.001^†^
Sex					<.001
Male	17,331 (54.4)	811 (48.9)	1,413 (20.1)	889 (44.8)	
Female	14,555 (45.6)	848 (51.1)	5,615 (79.9)	1,097 (55.2)	
Educational attainment (years)					<.001
≦6	7,175 (22.5)	412 (24.8)	2,816 (40.1)	567 (28.6)	
7–9	4,042 (12.7)	210 (12.7)	1,192 (17.0)	254 (12.8)	
≧10	20,669 (64.8)	1,037 (62.5)	3,020 (43.0)	1,165 (58.7)	
Low–income household	462 (1.5)	73 (4.4)	224 (3.2)	146 (7.4)	<.001
Living alone	284 (0.9)	523 (31.5)	1,793 (25.5)	398 (20.0)	<.001
BMI category					<.001
Underweight	1,151 (3.6)	88 (5.3)	306 (4.4)	136 (6.9)	
Normal weight	15,579 (48.9)	823 (49.6)	3,339 (47.5)	1,035 (52.1)	
Overweight	9,950 (31.2)	502 (30.3)	2,069 (29.4)	511 (25.7)	
Obesity	5,206 (16.3)	246 (14.8)	1,314 (18.7)	304 (15.3)	
Medication history					
Hypertension	14,038 (44.0)	643 (38.8)	3,594 (51.1)	772 (38.9)	<.001
Diabetes	4,199 (13.2)	218 (13.1)	967 (13.8)	240 (12.1)	0.251
Hyperlipidemia	3,853 (12.1)	175 (10.6)	912 (13.0)	204 (10.3)	0.002
Heart disease	5,717 (17.9)	303 (18.3)	1,598 (22.7)	322 (16.2)	<.001
Psychiatric history	1,930 (6.1)	144 (8.7)	565 (8.0)	102 (5.1)	<.001
Psychological distress	1,420 (4.5)	100 (6.0)	397 (5.7)	95 (4.8)	<.001
Current smoker	1,489 (4.7)	126 (7.6)	194 (2.8)	160 (8.1)	<.001
Occasional or frequent drinking	6,692 (21.0)	332 (20.0)	725 (10.3)	310 (15.6)	<.001

Note. SPMSQ, Short Portable Mental Status Questionnaire; AD8, Ascertain Dementia 8-item Informant Questionnaire; BMI, body mass index; married, married living together, and cohabiting; divorced, divorced and married living separately. The chi-square test was used for all variables, except for age, where ANOVA was used (indicated by †).

During the SPMSQ period (2005–2011), 1,314 participants (2.1%) screened positive for MCI over 3.7 ± 1.8 years (Table 3). After adjusting for confounders, never-married individuals showed increased risk (HR = 1.37, 95% CI: 1.08‒1.74), while divorced (HR = 1.32, 95% CI: 0.98‒1.78) and widowed (HR = 0.98, 95% CI: 0.85‒1.13) participants showed non-significant associations. In the AD8 period (2012–2016), 2,316 participants (5.4%) screened positive for MCI over 2.8 ± 1.1 years. In the AD8 period, the HR for never-married status was 0.60 (95% CI: 0.46–0.77), while divorced (HR = 1.03, 95% CI: 0.83–1.27) and widowed (HR = 1.04, 95% CI: 0.93–1.17) participants showed non-significant associations.

**Table 3:** Cox proportional hazards models of marital status predicting incident screen-positive cognitive impairment (MCI).

HR (95% CI)	Model 1	Model 2
SPMSQ period (2005–2011) (n = 61,304)		
Incident MCI, n (%)	1,314 (2.1)	
Follow-up duration, years (mean ± SD)	3.7 ± 1.8	
Marital status		
Married	Reference	Reference
Divorced	1.21 (0.91–1.62)	1.32 (0.98–1.78)
Widowed	0.95 (0.83–1.10)	0.98 (0.85–1.13)
Never married	1.62 (1.29–2.04)	1.37 (1.08–1.74)
AD8 period (2012–2016) (n = 42,559)		
Incident MCI, n (%)	2,316 (5.4)	
Follow-up duration (years, mean±SD)	2.8 ± 1.1	
Marital status		
Married	Reference	Reference
Divorced	1.13 (0.92–1.38)	1.03 (0.83–1.27)
Widowed	1.08 (0.97–1.20)	1.04 (0.93–1.17)
Never married	0.68 (0.54–0.85)	0.60 (0.46–0.77)

Notes: HR = hazard ratio; 95% CI = 95% confidence interval;

Model **1:** adjusted for age and sex

Model **2:** Model 1 plus education, BMI category, living alone, low-income status, hypertension, diabetes, dyslipidaemia, heart disease, stroke, psychiatric history, psychological distress, smoking, and alcohol consumption.

Table 4 presents stratified Cox regression analyses. During the SPMSQ period, the HR for never-married status was 1.89 (95% CI: 1.41–2.54) in males and the HR for divorced status was 1.78 (95% CI: 1.20–2.65) in females. During the AD8 period, the HR for never-married status was 0.66 in males and 0.53 in females. In the SPMSQ period, the HR for never-married status was 1.66 (95% CI: 1.19–2.30) among participants aged 65–79 years and was non-significant among those ≥80 years. In the AD8 period, the HR for never-married status was 0.42 (95% CI: 0.29–0.61) among participants aged 65–79 years and was non-significant among those ≥80 years. In the SPMSQ period, the HRs for divorced and never-married status were 1.61 and 1.48, respectively, among participants with <10 years of education, and were non-significant among those with ≥10 years of education. In the AD8 period, the HR for never-married status was 0.69 among participants with <10 years of education and 0.50 among those with ≥10 years of education.

**Table 4:** Subgroup Cox models of marital status and incident screen-positive cognitive impairment, stratified by sex, age, and education.

	Male	Female	Age 65–79 years old	Age ≧ 80 years old	Education <10 years	Education ≧10 years
Variable	HR (95% CI)	HR (95% CI)	HR (95% CI)	HR (95% CI)	HR (95% CI)	HR (95% CI)
SPMSQ period (2005–2011)						
Participants, n	31,980	29,324	48,994	12,310	29,145	32,159
Incident MCI, n (%)	596 (1.9)	718 (2.4)	711 (1.5)	603 (4.9)	881 (3.0)	433 (1.3)
Marital status						
Married	Reference	Reference	Reference	Reference	Reference	Reference
Divorced	1.01 (0.63–1.60)	1.78 (1.20–2.65)	1.19 (0.79–1.80)	1.50 (0.98–2.29)	1.61 (1.15–2.27)	0.86 (0.47–1.55)
Widowed	0.97 (0.74–1.27)	0.95 (0.80–1.12)	1.18 (0.97–1.42)	1.10 (0.90–1.33)	1.04 (0.88–1.22)	0.95 (0.72–1.26)
Never married	1.89 (1.41–2.54)	0.84 (0.54–1.30)	1.66 (1.19–2.30)	1.31 (0.92–1.85)	1.48 (1.12–1.95)	1.45 (0.90–2.35)
AD8 period (2012–2016) (n = 42,559)						
Participants, n	20,444	22,115	31,697	10,862	16,668	25,891
Incident MCI, n (%)	1,078 (5.3)	1,238 (5.6)	1,415 (4.6)	901 (8.3)	1,123 (6.7)	1,193 (4.6)
Marital status						
Married	Reference	Reference	Reference	Reference	Reference	Reference
Divorced	0.94 (0.68–1.29)	1.14 (0.85–1.51)	0.93 (0.71–1.23)	1.12 (0.79–1.57)	0.97 (0.71–1.32)	1.11 (0.83–1.50)
Widowed	0.95 (0.76–1.19)	1.11 (0.96–1.26)	1.21 (1.04–1.39)	1.09 (0.91–1.30)	1.02 (0.87–1.18)	1.15 (0.96–1.37)
Never married	0.66 (0.47–0.93)	0.53 (0.35–0.78)	0.42 (0.29–0.61)	0.91 (0.63–1.31)	0.69 (0.50–0.96)	0.50 (0.32–0.76)

Notes: HR = hazard ratio; 95% CI = 95% confidence interval; Adjusted for education, BMI group, living alone, low income, hypertension, diabetes, dyslipidemia, heart disease, stroke, psychiatric history, psychological distress, smoking, drinking.

Subgroup analyses examined potential effect modification across periods (Fig 2). During SPMSQ assessment, the marital status × sex interaction was significant (P = 0.008). During AD8 assessment, the marital status × age interaction (P < 0.001) and marital status × chronic disease interaction (P = 0.001) were significant. The interactions for education, BMI, living arrangement, and psychological distress were not significant. After Benjamini–Hochberg correction across the 14 interaction tests, three interactions remained significant: sex in the SPMSQ period (FDR-adjusted P = 0.036) and age and comorbidity in the AD8 period (both FDR-adjusted P = 0.004); the remaining interactions were not significant after adjustment (S3 Table).Sensitivity analyses stratified by follow-up duration and lag time are presented in Table 5. Never-married status showed non-significant, inconsistent associations during SPMSQ (HRs: 0.91–1.48) but consistent protection during AD8, strongest with <3 years follow-up (HR = 0.43, 95% CI: 0.29–0.64). The two-year lag analysis yielded an HR of 0.57 (95% CI: 0.44–0.75) for never-married status.

**Table 5:** Sensitivity analyses of Cox proportional hazards models of marital status predicting incident screen-positive cognitive impairment (MCI).

			Reference (married)		
	Follow-up Group	Incident MCI/total n	Divorced	Widowed	Never married
SPMSQ period (2005–2011) (n = 61,304)					
Model 1.1, HR (95% CI)	< 4 years	706/28,397	1.32 (0.87–2.00)	1.19 (0.99–1.43)	0.91 (0.68–1.23)
Model 1.2, HR (95% CI)	≥ 4 years	608/32,907	1.37 (0.89–2.10)	0.90 (0.73–1.11)	1.48 (0.99–2.20)
Model 1.3, HR (95% CI)	≥ 2 years lag	1,010/60,996	1.26 (0.89–1.77)	0.96 (0.81–1.13)	1.25 (0.94–1.67)
AD8 period (2012–2016) (n = 42,559)					
Model 2.1, HR (95% CI)	< 3 years	1,109/20,223	0.95 (0.71–1.28)	0.99 (0.84–1.17)	0.43 (0.29–0.64)
Model 2.2, HR (95% CI)	≥ 3 years	1,207/22,336	1.03 (0.75–1.41)	1.04 (0.89–1.22)	0.81 (0.57–1.14)
Model 2.3, HR (95% CI)	≥ 2 years lag	2,153/42,395	1.03 (0.82–1.29)	1.01 (0.89–1.14)	0.57 (0.44–0.75)

Note. HR = hazard ratio; 95% CI = 95% confidence interval; SPMSQ = Short Portable Mental Status Questionnaire; AD8 = Ascertain Dementia 8-item Informant Questionnaire; Model adjusted for age and sex, education, BMI group, living alone, low income, hypertension, diabetes, dyslipidemia, heart disease, stroke, psychiatric history, psychological distress, smoking, drinking.

“2 years lag” refers to excluding cases with MCI onset within 2 years from baseline.

## Discussion

This longitudinal study identified a reversal, not previously reported, in the association between marital status and incident MCI among never-married participants: increased risk (HR = 1.37, 95% CI: 1.08–1.74) during 2005–2011 versus reduced risk (HR = 0.60, 95% CI: 0.46–0.77) during 2012–2016. As detailed below and in the Limitations, this reversal coincides with a change in screening instrument, follow-up duration, and population composition across the two periods, and therefore may reflect measurement heterogeneity rather than a genuine temporal change in the underlying marital status–cognition relationship. If the latter interpretation holds, these findings would raise questions about the generalizability of prior assumptions about the role of marriage in cognitive health among aging populations and suggest that the relationship between marital status and cognitive outcomes may be more dynamic than previously recognized [14,25].

The observed protective associations among never-married individuals in the later period contrast with traditional models of successful aging, which often emphasize marriage as a cornerstone of optimal health [13,25,26]. However, the SPMSQ and AD8 periods differ not only in cognitive screening instrument but also in follow-up duration (mean 3.7 vs. 2.8 years) and in the composition of the study population across an 11-year span, and these dimensions are inherently confounded with calendar period in our design. In addition, because cognitive decline is time-dependent, the shorter mean follow-up in the AD8 period (2.8 years) relative to the SPMSQ period (3.7 years) may have disproportionately captured more rapid-onset cases while under-ascertaining the slower, more gradual cognitive decline that a longer observation window would detect; this near one-year difference in observation density adds a further source of imprecision—beyond simple confounding by calendar period—to any direct comparison of hazard ratios across the two periods. Our sensitivity analyses—including stratification by follow-up duration and a two-year lag analysis—were intended to address reverse causation and follow-up length, and the protective association among never-married participants persisted across these checks. This consistency is compatible with, but does not establish, a genuine temporal change, since the same checks cannot fully disentangle instrument effects, differential informant exposure, or secular shifts in the never-married population’s characteristics from a true change in the underlying marital status–cognition relationship. In particular, because the AD8 can be completed by either a co-residing informant or the participant, married individuals—who more often have a spouse available to observe subtle functional decline—may be more likely to be flagged as having cognitive impairment than never-married individuals, who more often rely on self-report; self-report is known to under-detect early cognitive decline relative to informant report, in part because of anosognosia (impaired insight into one’s own cognitive deficits) in the early stages of impairment. Given that never-married individuals in our cohort were markedly more likely to live alone (20.0% vs. 0.9% among married participants during the AD8 period), we consider this differential ascertainment by respondent type, rather than a true protective effect of never-married status, the more likely explanation for the reduced hazard observed during the AD8 period. The SPMSQ’s focus on crystallized cognitive abilities may have been more sensitive to traditional risk factors, while the AD8’s reliance on informant report makes it more susceptible to the differential ascertainment bias described above [27,28]; we therefore consider this instrument-driven detection bias, rather than any genuine cognitive advantage among never-married individuals, the more likely driver of the apparent reversal. We therefore present the temporal-shift interpretation as one plausible reading of the data rather than a firm conclusion, and emphasize that measurement and population heterogeneity remain fully viable alternative explanations (see Limitations). Indeed, because the transition from SPMSQ to AD8 coincides exactly with the boundary between the two calendar periods, and because the dataset does not include any interval in which both instruments were administered concurrently to allow direct calibration of one against the other, we cannot empirically distinguish a genuine secular change in the marital status–cognition relationship from an artifact of the instrument transition; given this lack of overlap-period calibration, the measurement-artifact explanation should be regarded as the more parsimonious interpretation and is prioritized over a social-change account in the conclusions we draw.

### Potential mechanisms

The hypothetical mechanisms outlined below assume that the observed reversal reflects, at least in part, a genuine temporal change rather than measurement heterogeneity between the SPMSQ and AD8 periods (see Limitations); we offer them only as illustrative possibilities for future testing, not as confirmed explanations, and we deliberately limit ourselves to the broadest plausible categories rather than an exhaustive list of pathways. During the earlier period, married individuals may have benefited from greater social support and health-behavior monitoring through spousal interaction, consistent with the stress-buffering hypothesis [13,29,30]; never-married individuals may have faced relative disadvantage in these domains. Any protective association observed for never-married status in the later period would be broadly consistent with a general hypothesis of lifelong independence fostering adaptive self-reliance and social-network management [31–33], though our study design cannot test this, nor distinguish it from the non-mechanistic, measurement-based explanations discussed above. We did not collect data on social network characteristics, decision-making autonomy, or technology use, and therefore do not pursue mechanism-specific speculation further beyond this general hypothesis; detailed assessment of these constructs is an important direction for future mixed-methods research (see Limitations).

### Sex and age interactions

Sex-stratified analyses showed elevated MCI risk among never-married men (HR = 1.89, 95% CI: 1.41–2.54) and divorced women (HR = 1.78, 95% CI: 1.20–2.65) during 2005–2011, broadly consistent with literature on sex differences in spousal dependence and the economic impact of marital dissolution [13,29,34,35]; both patterns reversed or attenuated during 2012–2016. The age interaction during the AD8 period—a protective association among younger-old adults (65–79 years: HR = 0.42) that was absent among the oldest-old—was the only subgroup interaction to remain significant after correction for multiple comparisons (S3 Table). Given the measurement heterogeneity discussed above, we do not attempt to attribute these subgroup patterns to specific mechanisms (e.g., shifting sex-role expectations or age-specific adaptive capacity); we report them descriptively as patterns warranting replication.During 2005–2011, adverse associations of non-married status were concentrated among lower-educated individuals, broadly consistent with cognitive reserve theory [36,37]; this education-stratified pattern was not observed during 2012–2016, when protective associations for never-married status appeared across educational strata. As with the sex and age patterns above, we report this descriptively rather than attributing it to a specific mechanism, given the measurement heterogeneity already discussed..

### Clinical and public health implications

Given the uncertainty surrounding the interpretation of our findings, we offer only tentative implications. Clinicians should be cautious about assuming marriage universally confers cognitive protection, and our findings do not, on their own, support specific intervention targets for never-married older adults; replication using consistent measurement approaches across periods is needed before practice or policy recommendations can be drawn. More broadly, our findings underscore the value of longitudinal monitoring of social determinants of cognitive health using consistent assessment methods, so that genuine temporal change can be distinguished from measurement artifact when evaluating how such associations evolve with societal change.

### Limitations

Several limitations warrant consideration. First, the observational design precludes definitive causal inferences, though comprehensive confounder adjustment and sensitivity analyses support the robustness of our findings. Second, the change in cognitive screening instruments between periods introduces measurement heterogeneity. While our sensitivity analyses may partially reflect temporal changes beyond measurement artifacts, we cannot entirely exclude the possibility that instrument characteristics contributed to observed differences [18,28]. The SPMSQ and AD8 assess different cognitive domains and rely on different informant sources, which may differentially capture cognitive changes across marital status groups [27,38]. Although our models adjusted for living arrangement (alone vs. with others), which partially captures the availability of a co-residing observer, the database did not record whether each AD8 assessment was completed by the participant or by a caregiver/informant, precluding direct testing of this hypothesis. Because married individuals are more likely to have a co-residing spousal informant who can detect subtle functional decline, whereas never-married individuals more often rely on self-report—a method prone to under-detection of early decline due to anosognosia—differential detection by respondent type, rather than a true protective effect, is the more likely explanation for the lower hazard observed among never-married participants during the AD8 period; this informant-related ascertainment bias should be considered alongside, and is not fully separable from, the broader measurement heterogeneity discussed above. The SPMSQ and AD8 periods differed not only in cognitive screening instrument but also in follow-up duration (mean 3.7 vs. 2.8 years), in the underlying data source, in how marital status was ascertained (a fixed baseline value in 2005–2011 vs. examination-updated values from 2012 onward), and—given the 11-year span of the cohort—in the demographic and socioeconomic composition of the never-married subgroup itself. The observed reversal among never-married participants should therefore be interpreted with caution, as calendar period, screening instrument, follow-up duration, and population composition are inherently confounded in this two-period design and cannot be fully disentangled with the available data.Because the two periods do not include an interval in which both instruments were administered concurrently, no direct calibration between SPMSQ and AD8 scores is possible, further limiting our ability to attribute the observed reversal to genuine temporal change rather than to the instrument transition itself. Future studies using consistent measurement tools across extended periods would provide more definitive evidence. Third, our focus on incident MCI rather than progression to dementia limits understanding of longer-term cognitive trajectories. Fourth, relatively short follow-up periods (median 3.2–3.8 years) may not capture the full spectrum of marital status effects on cognitive aging; moreover, because the AD8 period’s mean follow-up (2.8 years) was nearly one year shorter than that of the SPMSQ period (3.7 years), the two periods likely differ in observation density and in their relative sensitivity to rapid-onset versus slowly progressive cognitive decline, which adds further noise to any direct comparison of hazard ratios between periods beyond what Cox models, which adjust for time-at-risk, can fully account for. Fifth, the predominantly well-educated, health-conscious study population may limit generalizability to more diverse populations. Sixth, and most importantly, lack of detailed information on social networks, technology use, quality of marital relationships, and coping strategies limits our ability to identify precise mechanisms underlying the temporal shift. We did not measure digital technology engagement, social network characteristics, or psychosocial factors that may mediate the observed associations [12,34,39]. Future studies incorporating these variables through mixed-methods approaches would provide more definitive insights into causal pathways and enable testing of the hypothetical mechanisms proposed in this discussion. In addition, formal testing indicated some departure from the proportional hazards assumption for marital status, concentrated in the widowed and divorced/separated groups (whose associations were null); the never-married association central to our findings showed little or no such departure, and the reported hazard ratios should be interpreted as effects averaged over the follow-up period. Finally, because linked mortality data were incomplete, death could not be modeled as a competing event, and differential mortality by marital status could introduce informative censoring; our estimates therefore represent cause-specific hazards, and the small proportion of participants excluded for incomplete data (<2%) is unlikely to have biased the results. Beyond this, because the analytic cohort required at least two examinations, participants with a single examination were excluded; a comparison of baseline characteristics between the analytic cohort and these participants (S4 Table) showed comparable clinical comorbidity, sex, income, and body-mass distributions (all standardized mean differences < 0.10), with excluded participants being less educated and more often never-married and differing modestly on a few additional characteristics, all of which are adjusted for in our models. Residual selection toward a higher-functioning sample nonetheless cannot be excluded and would, if anything, bias the observed associations toward the null. Although the models adjusted for self-reported psychiatric medication use and for psychological distress measured by the BSRS-5, the database did not include a formal clinical or ICD-coded diagnosis of depression; residual confounding by undiagnosed or untreated depression therefore cannot be entirely excluded.

## Conclusion

This large longitudinal study of 103,863 community-dwelling older adults in Taiwan reveals a striking reversal in the association between marital status and incident screen-positive cognitive impairment: never-married status was associated with elevated MCI risk during the SPMSQ period (HR = 1.37, 95% CI: 1.08–1.74) but substantially reduced risk during the AD8 period (HR = 0.60, 95% CI: 0.46–0.77), with effect modification by sex, age, and comorbidity status identified across periods. These patterns persisted across sensitivity analyses incorporating follow-up stratification and a two-year lag analysis. The primary significance of these findings lies in demonstrating that cognitive assessment instrument choice can fundamentally alter observed marital status–cognition associations. Measurement heterogeneity and differential ascertainment bias—particularly the greater reliance on self-report among never-married individuals under the informant-based AD8—represent the most parsimonious explanation for the observed reversal, and should prompt researchers to revisit instrument-related sources of inconsistency in the broader marital status–cognition literature. This study contributes to the field in three ways. First, it provides one of the few systematic, large-scale comparisons of marital status–cognition associations across methodologically distinct screening instruments applied to the same source population. Second, its scope, duration (2005–2016), and comprehensive covariate adjustment offer a robust epidemiological platform for studying social determinants of cognitive health in a rapidly aging East Asian society. Third, by foregrounding measurement heterogeneity as a primary interpretive concern, it underscores the need for instrument standardization in future longitudinal aging research, and for caution when aggregating findings across studies using disparate cognitive assessment modalities.

## Supporting information

S1 Table Sensitivity analysis: marital status modeled as a time-varying covariate (AD8 period, 2012–2016). (DOCX)

S2 Table Test of the proportional hazards assumption for marital status. (DOCX)

S3 Table Effect modification of the marital status–cognitive impairment association, with multiplicity adjustment. (DOCX)

S4 Table Baseline characteristics of analyzed versus excluded (single-examination) participants. (DOCX)
