# Supplementary material for: Marital status and incident screen-positive cognitive impairment in a Taiwanese longitudinal cohort (2005–2016): Associations vary by cognitive screening instrument
Source: PLoS One. 2026 Oct 5;21(10):e0359778. doi: 10.1371/journal.pone.0359778 (PMC13637897; doi:10.1371/journal.pone.0359778)
Supplement: Supplementary file 1 [file pone.0359778.s001.docx]

Table S1. Sensitivity analysis: marital status modeled as a time-varying covariate (AD8 period, 2012–2016)

| **Marital status (vs. married)** | **Baseline HR (95% CI)** | **Time-varying HR (95% CI)** |
| --- | --- | --- |
| Divorced | 1.03 (0.83–1.27) | 1.10 (0.89–1.36) |
| Widowed | 1.04 (0.93–1.17) | 1.03 (0.92–1.16) |
| Never married | 0.60 (0.46–0.77) | 0.63 (0.49–0.81) |
| *Note.* Both models adjusted for age, sex, education, BMI category, living alone, low-income status, hypertension, diabetes, dyslipidemia, heart disease, stroke, psychiatric history, psychological distress, smoking, and alcohol consumption. The time-varying model treats marital status as a time-dependent covariate updated at each examination within a counting-process framework. A time-varying specification was not applied to the SPMSQ period (2005–2011) because marital status had no within-person variation in the source data for that period. | | |
