# Supplementary material for: Marital status and incident screen-positive cognitive impairment in a Taiwanese longitudinal cohort (2005–2016): Associations vary by cognitive screening instrument
Source: PLoS One. 2026 Oct 5;21(10):e0359778. doi: 10.1371/journal.pone.0359778 (PMC13637897; doi:10.1371/journal.pone.0359778)
Supplement: Supplementary file 2 [file pone.0359778.s002.docx]

**Table S2. Test of the proportional hazards assumption for marital status.**

| **Contrast (vs. married)** | **SPMSQ period** | **AD8 period** |
| --- | --- | --- |
| Divorced / separated | P = 0.088 | P = 0.002 |
| Widowed | P = 0.0005 | P < 0.0001 |
| Never married | P = 0.037 | P = 0.277 |
| **Overall (3 df)** | **P = 0.0008** | **P < 0.0001** |

*Note.* A marital status × log-time interaction was added to the fully adjusted model (Model 2); a significant interaction indicates departure from proportionality. Counting-process data; the cumulative-residual supremum (PROC PHREG ASSESS) test is not available in this setting, so PH was assessed via the time-interaction approach. SPMSQ n = 61,304 (1,314 events); AD8 n = 42,559 (2,316 events).
