# Supplementary material for: Marital status and incident screen-positive cognitive impairment in a Taiwanese longitudinal cohort (2005–2016): Associations vary by cognitive screening instrument
Source: PLoS One. 2026 Oct 5;21(10):e0359778. doi: 10.1371/journal.pone.0359778 (PMC13637897; doi:10.1371/journal.pone.0359778)
Supplement: Supplementary file 3 [file pone.0359778.s003.docx]

**Table S3. Effect modification of the marital status–cognitive impairment association, with multiplicity adjustment.**

| **Effect modifier** | **SPMSQ raw P** | **SPMSQ FDR P** | **AD8 raw P** | **AD8 FDR P** |
| --- | --- | --- | --- | --- |
| Sex | 0.008 | **0.036*** | 0.406 | 0.547 |
| Age (<80 / ≥80) | 0.245 | 0.547 | <0.001 | **0.004*** |
| Education | 0.401 | 0.547 | 0.135 | 0.379 |
| Comorbidity | 0.305 | 0.547 | <0.001 | **0.004*** |
| BMI | 0.692 | 0.745 | 0.983 | 0.983 |
| Living alone | 0.340 | 0.547 | 0.430 | 0.547 |
| Psychological distress | 0.076 | 0.265 | 0.632 | 0.737 |

*Note.* Each P-value is for the marital status × modifier interaction (joint 3-df Wald test). FDR-adjusted P-values use the Benjamini–Hochberg procedure across all 14 tests.

**Significant after FDR correction (adjusted P < 0.05). Benjamini–Hochberg applied jointly across all 14 interaction tests (7 modifiers × 2 periods).*
