# Supplementary material for: Marital status and incident screen-positive cognitive impairment in a Taiwanese longitudinal cohort (2005–2016): Associations vary by cognitive screening instrument
Source: PLoS One. 2026 Oct 5;21(10):e0359778. doi: 10.1371/journal.pone.0359778 (PMC13637897; doi:10.1371/journal.pone.0359778)
Supplement: Supplementary file 4 [file pone.0359778.s004.docx]

**Table S4. Baseline characteristics of analyzed versus excluded (single-examination) participants**

| **Characteristic** | **Analytic cohort** | **Excluded cohort** | **SMD** |
| --- | --- | --- | --- |
| **SPMSQ period (2005–2011) — analytic n = 61,304; excluded n = 44,254** | | | |
| Age, years (mean ± SD) | 74.0 ± 6.2 | 73.5 ± 7.3 | 0.074 |
| Male sex | 31,980 (52.2) | 21,397 (48.4) | 0.076 |
| **Marital status** |  |  | **0.264** |
| Married or cohabiting | 46,246 (75.4) | 30,308 (70.8) |  |
| Separated or divorced | 1,953 (3.2) | 1,477 (3.5) |  |
| Widowed | 10,362 (16.9) | 6,112 (14.3) |  |
| Never married | 2,743 (4.5) | 4,895 (11.4) |  |
| **Education** |  |  | **0.280** |
| Primary school or less | 20,185 (32.9) | 18,807 (46.3) |  |
| Junior high school | 8,960 (14.6) | 5,292 (13.0) |  |
| Senior high school or above | 32,159 (52.5) | 16,494 (40.6) |  |
| **BMI category** |  |  | 0.092 |
| Underweight | 2,184 (3.6) | 2,039 (4.7) |  |
| Normal | 28,200 (46.0) | 18,919 (43.4) |  |
| Overweight | 19,657 (32.1) | 13,455 (30.9) |  |
| Obese | 11,263 (18.4) | 9,162 (21.0) |  |
| Living alone | 3,993 (6.5) | 1,769 (4.0) | **0.113** |
| Low-income household | 2,885 (4.7) | 2,937 (6.6) | 0.083 |
| Hypertension | 25,186 (41.1) | 17,217 (38.9) | 0.045 |
| Diabetes | 7,375 (12.0) | 6,468 (14.6) | 0.076 |
| Dyslipidemia | 4,400 (7.2) | 2,719 (6.1) | 0.042 |
| Heart disease | 11,097 (18.1) | 7,285 (16.5) | 0.043 |
| Stroke | 566 (0.9) | 691 (1.6) | 0.058 |
| Psychiatric history | 2,197 (3.6) | 1,786 (4.0) | 0.024 |
| Psychological distress | 5,628 (9.2) | 4,968 (11.2) | 0.068 |
| Current smoking | 4,502 (7.3) | 4,682 (10.6) | **0.115** |
| Alcohol consumption | 12,228 (19.9) | 7,689 (17.4) | 0.065 |
|  |  |  |  |
| **AD8 period (2012–2016) — analytic n = 42,559; excluded n = 32,700** | | | |
| Age, years (mean ± SD) | 75.0 ± 6.7 | 73.6 ± 7.9 | **0.185** |
| Male sex | 20,444 (48.0) | 14,779 (45.2) | 0.057 |
| **Marital status** |  |  | **0.225** |
| Married or cohabiting | 31,886 (74.9) | 22,145 (67.8) |  |
| Separated or divorced | 1,659 (3.9) | 1,890 (5.8) |  |
| Widowed | 7,028 (16.5) | 5,441 (16.7) |  |
| Never married | 1,986 (4.7) | 3,183 (9.8) |  |
| **Education** |  |  | **0.163** |
| Primary school or less | 10,970 (25.8) | 10,533 (33.1) |  |
| Junior high school | 5,698 (13.4) | 4,096 (12.9) |  |
| Senior high school or above | 25,891 (60.8) | 17,194 (54.0) |  |
| **BMI category** |  |  | 0.084 |
| Underweight | 1,681 (4.0) | 1,329 (4.1) |  |
| Normal | 20,776 (48.8) | 14,726 (45.6) |  |
| Overweight | 13,032 (30.6) | 9,953 (30.8) |  |
| Obese | 7,070 (16.6) | 6,307 (19.5) |  |
| Living alone | 2,998 (7.0) | 1,812 (5.5) | 0.062 |
| Low-income household | 905 (2.1) | 779 (2.4) | 0.017 |
| Hypertension | 19,047 (44.8) | 13,881 (42.5) | 0.046 |
| Diabetes | 5,624 (13.2) | 5,042 (15.4) | 0.063 |
| Dyslipidemia | 5,144 (12.1) | 3,572 (10.9) | 0.037 |
| Heart disease | 7,940 (18.7) | 6,067 (18.6) | 0.003 |
| Stroke | 404 (0.9) | 459 (1.4) | 0.042 |
| Psychiatric history | 2,741 (6.4) | 2,156 (6.6) | 0.006 |
| Psychological distress | 2,012 (4.7) | 3,129 (9.6) | **0.189** |
| Current smoking | 1,969 (4.6) | 2,133 (6.5) | 0.083 |
| Alcohol consumption | 8,059 (18.9) | 6,092 (18.7) | 0.007 |

*Note.* Values are n (%) unless otherwise indicated; for the excluded cohort, percentages are calculated among participants with non-missing data for each variable, whereas the analytic cohort has complete covariate data by design. The excluded cohort comprises participants excluded for a single examination, as shown in Figure 1. SMD = standardized mean difference (continuous variables, difference in means divided by the pooled standard deviation; categorical variables, the multinomial standardized difference); |SMD| < 0.10 denotes a negligible between-group difference, and values ≥ 0.10 are shown in bold. SPMSQ = Short Portable Mental Status Questionnaire; AD8 = Ascertain Dementia 8-item Informant Questionnaire; BMI = body mass index; SD = standard deviation.
