# Supplementary material for: Marital status and incident screen-positive cognitive impairment in a Taiwanese longitudinal cohort (2005–2016): Associations vary by cognitive screening instrument
Source: PLoS One. 2026 Oct 5;21(10):e0359778. doi: 10.1371/journal.pone.0359778 (PMC13637897; doi:10.1371/journal.pone.0359778)
Supplement: Supplementary file 5 [file pone.0359778.s006.docx]

**RESPONSE TO ACADEMIC EDITOR’S JOURNAL REQUIREMENTS**

**JOURNAL REQUIREMENT 1:**

Please ensure that your manuscript meets PLOS ONE’s style requirements, including those for file naming.

**RESPONSE TO JOURNAL REQUIREMENT 1:**

We thank the Editor for this reminder. We have reviewed the revised manuscript against the PLOS ONE style templates (the main-body sample and the title/authors/affiliations sample) and confirmed that the title page, section headings, in-text citation order for figures and tables, double line spacing, and continuous line numbering conform to PLOS ONE’s formatting requirements. We have also reviewed the naming of our manuscript and accompanying files to ensure compliance with PLOS ONE’s file-naming conventions.

**JOURNAL REQUIREMENT 2:**

The ethical approval number(s) listed in the manuscript and/or submission metadata does not match the approval number on the ethical approval document you provided. Please ensure that all approval numbers are correct.

**RESPONSE TO JOURNAL REQUIREMENT 2:**

We apologize for this discrepancy and thank the Editor for identifying it. We have verified the ethics approval number against the official approval certificate issued by the Research Ethics Committee of Taipei City Hospital and confirmed that the correct number is TCHIRB-11303014-E. This number is now stated correctly and consistently in the Methods section of the manuscript (Study population subsection), and we have corrected the corresponding field in the online submission metadata so that it matches the approval document and the manuscript text.

**JOURNAL REQUIREMENT 3:**

Thank you for stating the following financial disclosure: “This study was supported by the Taipei City Hospital, Taiwan (TPCH-114-06).” Please state what role the funders took in the study. If the funders had no role, please state: “The funders had no role in study design, data collection and analysis, decision to publish, or preparation of the manuscript.” If this statement is not correct you must amend it as needed. Please include this amended Role of Funder statement in your cover letter; we will change the online submission form on your behalf.

**RESPONSE TO JOURNAL REQUIREMENT 3:**

We thank the Editor for this request. The funder, Taipei City Hospital, provided access to the Taipei City Elderly Health Examination Database used in this study but had no other role in the research. We have amended our Role of Funder statement accordingly and included it in our cover letter as follows: “This study was supported by the Taipei City Hospital, Taiwan (TPCH-114-06). The funder provided access to the Taipei City Elderly Health Examination Database for this study. The funder had no role in study design, data collection and analysis, decision to publish, or preparation of the manuscript.” We kindly ask the editorial office to update the online submission form accordingly.

**JOURNAL REQUIREMENT 4:**

Please note that funding information should not appear in any section or other areas of your manuscript. We will only publish funding information present in the Funding Statement section of the online submission form. Please remove any funding-related text from the manuscript.

**RESPONSE TO JOURNAL REQUIREMENT 4:**

We have removed all funding-related text from the body of the manuscript. The Role of Funder statement now appears only in our cover letter for entry into the Funding Statement section of the online submission form, in line with PLOS ONE policy.

**JOURNAL REQUIREMENT 5:**

We note that you have indicated that there are restrictions to data sharing for this study. PLOS only allows data to be available upon request if there are legal or ethical restrictions on sharing data publicly. Before we proceed with your manuscript, please address the following prompts: a) If there are ethical or legal restrictions on sharing a de-identified data set, please explain them in detail and who has imposed them. Please also provide contact information for a data access committee, ethics committee, or other institutional body to which data requests may be sent. b) If there are no restrictions, please upload the minimal anonymized data set necessary to replicate your study findings to a stable, public repository and provide us with the relevant URLs, DOIs, or accession numbers.

**RESPONSE TO JOURNAL REQUIREMENT 5:**

We confirm that legal and ethical restrictions apply to public sharing of the de-identified data set used in this study. The Taipei City Elderly Health Examination Database is a government-administered public health database owned by Taipei City Hospital. Access restrictions are imposed under Taiwan’s Personal Data Protection Act and by the Research Ethics Committee of Taipei City Hospital, which oversees data access to protect participant privacy; these restrictions are unrelated to any competing interests of the authors. Other researchers may access the database through the same process used by the authors, by submitting a formal data access application to Taipei City Hospital. For guidance on the application procedure, researchers may contact Taipei City Hospital Research Ethics Committee (Email: hrp@tpech.gov.tw). We have incorporated this information into the Data Availability Statement of the revised manuscript and would welcome the Editor’s assistance in updating the online Data Availability Statement accordingly.

**JOURNAL REQUIREMENT 6:**

Your ethics statement should only appear in the Methods section of your manuscript. If your ethics statement is written in any section besides the Methods, please move it to the Methods section and delete it from any other section. Please ensure that your ethics statement is included in your manuscript, as the ethics statement entered into the online submission form will not be published alongside your manuscript.

**RESPONSE TO JOURNAL REQUIREMENT 6:**

We confirm that the ethics statement—approval by the Institutional Review Board of Taipei City Hospital, the exemption from informed consent, and adherence to the principles of the Declaration of Helsinki—appears only once in the manuscript, in the Study population subsection of the Methods section, and does not appear in any other section (e.g., Abstract, Acknowledgments).

**JOURNAL REQUIREMENT 7:**

We notice that your supplementary figures are uploaded with the file type ‘Figure’. Please amend the file type to ‘Supporting Information’. Please ensure that each Supporting Information file has a legend listed in the manuscript after the references list.

**RESPONSE TO JOURNAL REQUIREMENT 7:**

We thank the Editor for noting this. We have amended the file type for our Supporting Information files (S1–S4 Table) from ‘Figure’ to ‘Supporting Information’ in Editorial Manager, and we have added a Supporting Information section, listing the legend for each Supporting Information file (S1 Table, S2 Table, S3 Table, and S4 Table), immediately after the reference list in the revised manuscript.

**JOURNAL REQUIREMENT 8:**

If the reviewer comments include a recommendation to cite specific previously published works, please review and evaluate these publications to determine whether they are relevant and should be cited. There is no requirement to cite these works unless the editor has indicated otherwise.

**RESPONSE TO JOURNAL REQUIREMENT 8:**

We have reviewed the works referenced in the reviewers’ comments below for relevance to our manuscript. Where we judged a recommended publication to be directly relevant, we have evaluated and, where appropriate, incorporated it into our reference list and discussion (see, for example, our response to Reviewer #2, Comment 1, regarding the addition of reference #19 on reverse causality). We did not identify any additional reviewer-recommended publications that were directly relevant but omitted from our revision.

**REVIEWER 1 COMMENTS**

**COMMENT 1:**

The comparison between the SPMSQ and AD8 periods is difficult to interpret. The two periods differ not only in calendar time but also in cognitive screening instrument, follow-up duration, and population composition. Therefore, the apparent reversal of the association among never-married participants may reflect measurement heterogeneity rather than a true temporal change in the marital status–cognition relationship.

**RESPONSE TO COMMENT 1:**

We thank the reviewer for raising this important point, and we agree. On reflection, our original Discussion and Conclusion framed the observed reversal in the never-married association as a “temporal shift” more confidently than a two-period observational comparison of this kind can support, given that calendar period, screening instrument, follow-up duration (mean 3.7 vs. 2.8 years), and population composition all change simultaneously between 2005–2011 and 2012–2016 and cannot be fully disentangled with the available data. We have revised the manuscript throughout—Abstract, Discussion, and Conclusion—to explicitly state that measurement heterogeneity is a fully plausible alternative explanation for the observed reversal, on equal footing with a genuine temporal change, and we have reframed our speculative mechanistic discussion (societal/technological hypotheses, sex- and age-stratified patterns, and the education-stratified pattern) as conditional on the reversal being genuine rather than an artifact of period-instrument confounding. We believe these revisions bring the strength of our interpretive language into alignment with what the study design can actually support, and we are grateful to the reviewer for prompting this clarification.

**Revised text:**

**Abstract, Conclusions (p. 2–3, lines 37–43):**

“Associations between marital status and incident MCI differed across assessment periods that varied in screening instrument, follow-up duration, and population composition; this measurement heterogeneity, rather than a confirmed temporal change, may account for the reversal observed among never-married participants. These findings suggest that interpretation of marital status–cognition associations should be considered alongside the measurement approach and study period under which they were derived.”

**Discussion, opening paragraph (p. 25, lines 309–319):**

“This longitudinal study identified a reversal, not previously reported, in the association between marital status and incident MCI among never-married participants: increased risk (HR=1.37, 95% CI: 1.08-1.74) during 2005-2011 versus reduced risk (HR=0.60, 95% CI: 0.46-0.77) during 2012-2016. As detailed below and in the Limitations, this reversal coincides with a change in screening instrument, follow-up duration, and population composition across the two periods, and therefore may reflect measurement heterogeneity rather than a genuine temporal change in the underlying marital status–cognition relationship. If the latter interpretation holds, these findings would raise questions about the generalizability of prior assumptions about the role of marriage in cognitive health among aging populations and suggest that the relationship between marital status and cognitive outcomes may be more dynamic than previously recognized [14, 25].”

**Discussion, second paragraph (p. 25–27, lines 320–362):**

*(This paragraph now also incorporates the revisions made in response to Comments 2, 9, and 11 below; we reproduce the full, current paragraph here so that it can be located verbatim.)*

“The observed protective associations among never-married individuals in the later period contrast with traditional models of successful aging, which often emphasize marriage as a cornerstone of optimal health [13, 25, 26]. However, the SPMSQ and AD8 periods differ not only in cognitive screening instrument but also in follow-up duration (mean 3.7 vs. 2.8 years) and in the composition of the study population across an 11-year span, and these dimensions are inherently confounded with calendar period in our design. In addition, because cognitive decline is time-dependent, the shorter mean follow-up in the AD8 period (2.8 years) relative to the SPMSQ period (3.7 years) may have disproportionately captured more rapid-onset cases while under-ascertaining the slower, more gradual cognitive decline that a longer observation window would detect; this near one-year difference in observation density adds a further source of imprecision—beyond simple confounding by calendar period—to any direct comparison of hazard ratios across the two periods. Our sensitivity analyses—including stratification by follow-up duration and a two-year lag analysis—were intended to address reverse causation and follow-up length, and the protective association among never-married participants persisted across these checks. This consistency is compatible with, but does not establish, a genuine temporal change, since the same checks cannot fully disentangle instrument effects, differential informant exposure, or secular shifts in the never-married population’s characteristics from a true change in the underlying marital status–cognition relationship. In particular, because the AD8 can be completed by either a co-residing informant or the participant, married individuals—who more often have a spouse available to observe subtle functional decline—may be more likely to be flagged as having cognitive impairment than never-married individuals, who more often rely on self-report; self-report is known to under-detect early cognitive decline relative to informant report, in part because of anosognosia (impaired insight into one’s own cognitive deficits) in the early stages of impairment. Given that never-married individuals in our cohort were markedly more likely to live alone (20.0% vs. 0.9% among married participants during the AD8 period), we consider this differential ascertainment by respondent type, rather than a true protective effect of never-married status, the more likely explanation for the reduced hazard observed during the AD8 period. The SPMSQ’s focus on crystallized cognitive abilities may have been more sensitive to traditional risk factors, while the AD8’s reliance on informant report makes it more susceptible to the differential ascertainment bias described above [28, 29]; we therefore consider this instrument-driven detection bias, rather than any genuine cognitive advantage among never-married individuals, the more likely driver of the apparent reversal. We therefore present the temporal-shift interpretation as one plausible reading of the data rather than a firm conclusion, and emphasize that measurement and population heterogeneity remain fully viable alternative explanations (see Limitations). Indeed, because the transition from SPMSQ to AD8 coincides exactly with the boundary between the two calendar periods, and because the dataset does not include any interval in which both instruments were administered concurrently to allow direct calibration of one against the other, we cannot empirically distinguish a genuine secular change in the marital status–cognition relationship from an artifact of the instrument transition; given this lack of overlap-period calibration, the measurement-artifact explanation should be regarded as the more parsimonious interpretation and is prioritized over a social-change account in the conclusions we draw.”

**Discussion, Potential Mechanisms (p. 27, lines 364–379):**

*(This section was further condensed in response to Comment 7 below; the text below is the final version, and is identical to the text reproduced under Comment 7.)*

“The hypothetical mechanisms outlined below assume that the observed reversal reflects, at least in part, a genuine temporal change rather than measurement heterogeneity between the SPMSQ and AD8 periods (see Limitations); we offer them only as illustrative possibilities for future testing, not as confirmed explanations, and we deliberately limit ourselves to the broadest plausible categories rather than an exhaustive list of pathways. During the earlier period, married individuals may have benefited from greater social support and health-behavior monitoring through spousal interaction, consistent with the stress-buffering hypothesis [13, 31, 32]; never-married individuals may have faced relative disadvantage in these domains. Any protective association observed for never-married status in the later period would be broadly consistent with a general hypothesis of lifelong independence fostering adaptive self-reliance and social-network management [33-35] , though our study design cannot test this, nor distinguish it from the non-mechanistic, measurement-based explanations discussed above. We did not collect data on social network characteristics, decision-making autonomy, or technology use, and therefore do not pursue mechanism-specific speculation further beyond this general hypothesis; detailed assessment of these constructs is an important direction for future mixed-methods research (see Limitations).”

**Discussion, Sex and Age Interactions (p. 27–28, lines 380–390):**

*(Final, condensed version — identical to the text reproduced under Comment 7.)*

“Sex-stratified analyses showed elevated MCI risk among never-married men (HR=1.89, 95% CI: 1.41-2.54) and divorced women (HR=1.78, 95% CI: 1.20-2.65) during 2005-2011, broadly consistent with literature on sex differences in spousal dependence and the economic impact of marital dissolution [13, 31, 49, 51]; both patterns reversed or attenuated during 2012-2016. The age interaction during the AD8 period—a protective association among younger-old adults (65-79 years: HR=0.42) that was absent among the oldest-old—was the only subgroup interaction to remain significant after correction for multiple comparisons (S3 Table). Given the measurement heterogeneity discussed above, we do not attempt to attribute these subgroup patterns to specific mechanisms (e.g., shifting sex-role expectations or age-specific adaptive capacity); we report them descriptively as patterns warranting replication.”

**Discussion, education-stratified paragraph (p. 28, lines 390–396):**

*(Final, condensed version — identical to the text reproduced under Comment 7.)*

“During 2005-2011, adverse associations of non-married status were concentrated among lower-educated individuals, broadly consistent with cognitive reserve theory [53, 54]; this education-stratified pattern was not observed during 2012-2016, when protective associations for never-married status appeared across educational strata. As with the sex and age patterns above, we report this descriptively rather than attributing it to a specific mechanism, given the measurement heterogeneity already discussed.”

**Discussion, Limitations (p. 29–30, lines 426–434):**

“The SPMSQ and AD8 periods differed not only in cognitive screening instrument but also in follow-up duration (mean 3.7 vs. 2.8 years), in the underlying data source, in how marital status was ascertained (a fixed baseline value in 2005–2011 vs. examination-updated values from 2012 onward), and—given the 11-year span of the cohort—in the demographic and socioeconomic composition of the never-married subgroup itself. The observed reversal among never-married participants should therefore be interpreted with caution, as calendar period, screening instrument, follow-up duration, and population composition are inherently confounded in this two-period design and cannot be fully disentangled with the available data.”

**Conclusion (p. 31–32, lines 476–482):**

“This longitudinal study identified a reversal in the association between marital status and incident MCI, with never-married status associated with increased risk during the earlier period but reduced risk during the later period. Because the SPMSQ and AD8 periods differ in screening instrument, follow-up duration, and population composition, measurement heterogeneity remains a fully plausible alternative explanation for this reversal, and our findings should not be read as establishing a true temporal change in the marital status–cognition relationship.”

**COMMENT 2:**

The AD8 assessment may introduce differential reporting bias by marital status. Because AD8 can be completed by either an informant or the participant, married individuals may be more likely to have spouse-based observations, whereas never-married individuals may rely more often on self-report or less frequent observers. This could lead to differential detection of cognitive decline and may partly explain the apparently protective association of never-married status in the AD8 period.

**RESPONSE TO COMMENT 2:**

We thank the reviewer for highlighting this important alternative explanation. We agree that differential ascertainment by respondent type is a plausible mechanism that could generate, rather than merely accompany, the apparently protective association observed for never-married status during the AD8 period. The AD8 can indeed be completed by either a co-residing informant or the participant; married individuals more often have a spouse available to detect subtle functional decline, whereas never-married individuals more often rely on self-report, and self-report is known to under-detect early cognitive decline relative to informant report. If this differential detection occurred systematically by marital status, it could by itself produce a lower observed hazard for never-married participants without any true difference in underlying cognitive risk. We have clarified in the Methods that our database did not record which respondent type was used for each AD8 assessment, precluding direct stratification by informant type, and we have expanded the Discussion and Limitations to explicitly present this informant-related ascertainment bias as a plausible explanation for the observed pattern, alongside the broader measurement heterogeneity already discussed in response to Comment 1. We note that our models did adjust for living arrangement (alone vs. with others), which partially captures informant availability, but this is an imperfect proxy and cannot fully rule out residual bias from differential respondent type.

**Revised text:**

**Methods, Measures of Cognitive Impairment (p. 10, lines 148–153):**

“This assessment tool offers flexibility in administration, allowing completion through either caregiver observation or individual self-evaluation; however, the database did not record which respondent type (self vs. caregiver/informant) was used for each assessment, precluding direct stratification by informant type. Both the SPMSQ and AD8 used in this study were traditional Chinese versions that have been well validated in the Taiwanese population.”

**Discussion, second paragraph (p. 26, lines 338–348):**

*(This sentence was further sharpened in response to Comment 12 below; the text below is the final version, identical to the text reproduced under Comment 12.)*

“In particular, because the AD8 can be completed by either a co-residing informant or the participant, married individuals—who more often have a spouse available to observe subtle functional decline—may be more likely to be flagged as having cognitive impairment than never-married individuals, who more often rely on self-report; self-report is known to under-detect early cognitive decline relative to informant report, in part because of anosognosia (impaired insight into one’s own cognitive deficits) in the early stages of impairment. Given that never-married individuals in our cohort were markedly more likely to live alone (20.0% vs. 0.9% among married participants during the AD8 period), we consider this differential ascertainment by respondent type, rather than a true protective effect of never-married status, the more likely explanation for the reduced hazard observed during the AD8 period.”

**Discussion, Limitations (p. 29, lines 414–426):**

*(This sentence was further strengthened in response to Comment 12 below; the text below is the final version, identical to the text reproduced under Comment 12.)*

“The SPMSQ and AD8 assess different cognitive domains and rely on different informant sources, which may differentially capture cognitive changes across marital status groups [28, 30]. Although our models adjusted for living arrangement (alone vs. with others), which partially captures the availability of a co-residing observer, the database did not record whether each AD8 assessment was completed by the participant or by a caregiver/informant, precluding direct testing of this hypothesis. Because married individuals are more likely to have a co-residing spousal informant who can detect subtle functional decline, whereas never-married individuals more often rely on self-report—a method prone to under-detection of early decline due to anosognosia—differential detection by respondent type, rather than a true protective effect, is the more likely explanation for the lower hazard observed among never-married participants during the AD8 period; this informant-related ascertainment bias should be considered alongside, and is not fully separable from, the broader measurement heterogeneity discussed above.”

**COMMENT 3:**

SPMSQ and AD8 are screening tools rather than clinical diagnostic instruments for MCI. The term “incident MCI” may overstate diagnostic certainty. The authors should consider using “screen-positive cognitive impairment” or “probable cognitive impairment” unless clinical diagnostic confirmation is available.

**RESPONSE TO COMMENT 3:**

We thank the reviewer for this valuable point and agree that “incident MCI,” as originally framed, could be read as implying a clinical diagnosis that our data do not support. Cognitive status in this study was ascertained solely through standardized cutoffs on the SPMSQ and AD8, both validated screening instruments, with no clinical or neuropsychological confirmation available in the database. We have therefore revised the manuscript title, Abstract, Methods, and the three relevant table titles to describe the outcome as “screen-positive cognitive impairment.” Because the abbreviation “MCI” is used extensively throughout the Results, Discussion, and all tables (more than 50 instances), we retained this abbreviation for readability and consistency with prior literature using these instruments, but we have added an explicit statement in the Methods, at first use, clarifying that MCI in this paper denotes screen-positive status rather than a clinical diagnosis. We have also revised a sentence in the Methods that referred to MCI cases as “diagnosed,” replacing it with “identified” to avoid the same overstatement of certainty. We believe these changes accurately convey the screening-based nature of our outcome measure while preserving the readability of the manuscript.

**Revised text:**

**Title (p. 1, lines 1–3):**

“Marital status and incident screen-positive cognitive impairment in a Taiwanese longitudinal cohort (2005–2016): Associations vary by cognitive screening instrument”

**Abstract, Background (p. 2, lines 21–24):**

“This study examined whether associations between marital status and incident screen-positive cognitive impairment (MCI) differ by screening instrument and population subgroups in a large longitudinal cohort of community-dwelling older adults in Taiwan.”

**Methods, Measures of cognitive function (p. 9, lines 131–136):**

“In this study, screen-positive cognitive impairment (hereafter referred to as MCI for brevity and consistency with prior literature using these instruments) was ascertained using the SPMSQ and AD8 questionnaires. As both the SPMSQ and AD8 are validated screening tools rather than clinical diagnostic instruments, the resulting classification reflects screen-positive status based on a standardized cutoff rather than a clinical diagnosis of mild cognitive impairment or dementia.”

**Methods, Sensitivity analyses (p. 15, lines 217–219):**

“Sensitivity analyses assessed robustness: (1) stratification by median follow-up duration (<4 vs. ≥4 years for SPMSQ; <3 vs. ≥3 years for AD8); (2) exclusion of screen-positive MCI cases identified within the first 2 years to mitigate reverse causality.”

**Table 3 title (p. 20, lines 263–264):**

“Table 3. Cox proportional hazards models of marital status predicting incident screen-positive cognitive impairment (MCI)”

**Table 4 title (p. 22, line 283):**

“Table 4. Subgroup Cox models of marital status and incident screen-positive cognitive impairment, stratified by sex, age, and education”

**Table 5 title (p. 24, line 301):**

“Table 5. Sensitivity analyses of Cox proportional hazards models of marital status predicting incident screen-positive cognitive impairment (MCI)”

**COMMENT 4:**

Marital status was treated as a fixed baseline exposure. Changes in marital status during follow-up, including widowhood, remarriage, separation, or changes in living arrangements, were not accounted for. This may introduce exposure misclassification, particularly in an older cohort.

**RESPONSE TO COMMENT 4:**

We thank the reviewer for this valuable suggestion. Marital status was self-reported at examination. Marital status was ascertained at baseline, and within-person updates were available only in the later study period (2012–2016); in the earlier period (2005–2011) marital status was recorded as a baseline value without within-person variation. For the SPMSQ period we therefore used baseline marital status, as the source data do not support a time-varying specification for this period. For the AD8 period, where within-person updates were available, we re-fitted the model treating marital status as a time-varying covariate within a counting-process framework. The estimates were materially unchanged: never-married status remained significantly protective (HR = 0.63, 95% CI 0.49–0.81, vs. baseline HR = 0.60, 0.46–0.77), and divorced and widowed status remained non-significant. These results indicate that exposure misclassification from marital transitions does not account for our findings. We have clarified the Methods accordingly and added a limitation noting that the two periods were derived from data sources with differing marital-status ascertainment.

**Revised text:**

**Discussion, Limitations (p. 29–30, lines 426–434):**

*(This is the same sentence already revised, and further expanded, in response to Comment 1 above; we reproduce the final version here so it matches the manuscript exactly.)*

“The SPMSQ and AD8 periods differed not only in cognitive screening instrument but also in follow-up duration (mean 3.7 vs. 2.8 years), in the underlying data source, in how marital status was ascertained (a fixed baseline value in 2005–2011 vs. examination-updated values from 2012 onward), and—given the 11-year span of the cohort—in the demographic and socioeconomic composition of the never-married subgroup itself. The observed reversal among never-married participants should therefore be interpreted with caution, as calendar period, screening instrument, follow-up duration, and population composition are inherently confounded in this two-period design and cannot be fully disentangled with the available data.”

**COMMENT 5 and 6:**

- The study lacks data on marital quality, loneliness, social network size, frequency of social participation, caregiver availability, technology use, and cognitive activities. These omissions limit mechanistic interpretation and raise the possibility of residual confounding.
- The authors should report whether proportional hazards assumptions were tested, how competing risk of death was handled, how missing data were addressed, and whether multiple testing was considered in subgroup and interaction analyses.

**RESPONSE TO COMMENT 5 and 6:**

We thank the reviewer for raising these four important methodological points, which we address individually below. The corresponding changes have been incorporated into the Methods, Results, Discussion, and Supplementary Material.

**(a) Proportional hazards (PH) assumption.** We thank the reviewer for prompting this check. Because our models use a counting-process data structure with time-updated covariates, the cumulative-residual supremum test (PROC PHREG ASSESS) is not available in this setting; we therefore assessed PH by adding an interaction between marital status and log time to the fully adjusted model and testing its significance (Therneau & Grambsch, 2000). The overall interaction was statistically significant in both periods (SPMSQ P = 0.0008; AD8 P < 0.0001). We interpret this cautiously, because with 1,314 and 2,316 events these tests are highly powered and detect even slight departures from proportionality. Importantly, the departure was concentrated in the widowed and divorced/separated contrasts—both of which were null in the main analysis—whereas the never-married contrast underlying our principal findings showed only a modest departure in the SPMSQ period (P = 0.037) and none in the AD8 period (P = 0.277). Under non-proportionality, the Cox hazard ratio estimates a well-defined average effect over the observed follow-up, which remains an appropriate summary of the association (Xu & O’Quigley, 2000); the estimates were materially unchanged when evaluated at the mean follow-up time. These results are now reported in the Methods and in S2 Table, and a corresponding limitation has been added to the Discussion.

**(b) Competing risk of death.** We thank the reviewer for this point. We did not model death as a competing risk. Linked mortality data were available only through 2012, which precludes a complete competing-risks (subdistribution hazard) analysis over the full study window and, in particular, for the AD8 period (2012–2016). Moreover, our research question is etiologic, for which the cause-specific hazard estimated by the Cox model is the appropriate estimand (Lau, Cole & Gange, 2009; Austin, Lee & Fine, 2016). Cognitive status was ascertained at repeated health examinations, and participants who did not screen positive were censored at their last attended examination, with death being one of several reasons for non-attendance. We have clarified the censoring scheme in the Methods and added a limitation noting that, because death and other forms of loss to follow-up could not be fully disentangled and mortality may differ by marital status, informative censoring cannot be excluded and our estimates should be interpreted as cause-specific hazards.

**(c) Missing data.** We thank the reviewer for raising this. We used a complete-case analysis. Participants with incomplete questionnaire data on any model covariate were excluded (n = 1,185 [1.9%] in the SPMSQ period; n = 709 [1.6%] in the AD8 period), as shown in the participant flow diagram (Fig 1) and now stated explicitly in the Methods. Because missingness was below 2% in both periods, complete-case analysis is unlikely to have materially biased the results.

**(d) Multiple testing.** We thank the reviewer for this suggestion. The subgroup and interaction analyses were exploratory. We have applied a Benjamini–Hochberg false discovery rate (FDR) correction across all 14 interaction tests (7 effect modifiers × 2 periods). Only three interactions remained significant after correction: sex in the SPMSQ period (FDR-adjusted P = 0.036) and age and comorbidity in the AD8 period (both FDR-adjusted P = 0.004). These correspond precisely to the only effect modifications interpreted in the manuscript; none of the remaining interactions survived correction. The full set of raw and FDR-adjusted P-values is now provided in S3 Table, and we have revised the Results and Discussion to describe these analyses as exploratory and to interpret them in light of this multiplicity adjustment.

**Revised text:**

**Methods, Statistical analyses (p. 12–13, lines 199–204):**

“Participants who did not screen positive were censored at their last attended examination, with death treated as censoring. Because linked mortality data were available only through 2012, a competing-risks analysis was not performed, and hazard ratios are reported as cause-specific hazards. Analyses were restricted to participants with complete data on all model covariates (complete-case analysis); the numbers excluded for incomplete questionnaire data are shown in Fig 1.”

**Methods, Statistical analyses (p. 15, lines 214–217):**

“Because effect modification was examined across multiple subgroups, P-values for all marital status × modifier interactions were adjusted for multiple comparisons using the Benjamini–Hochberg false discovery rate across the full set of 14 interaction tests (S3 Table).”

**Methods, Statistical analyses (p. 15, lines 219–222):**

“The proportional hazards assumption was evaluated by adding an interaction between marital status and log time to the fully adjusted model; because the counting-process formulation precludes the cumulative-residual supremum test, this time-interaction approach was used (S2 Table).”

**Results (p. 23, lines 291–295):**

“After Benjamini–Hochberg correction across the 14 interaction tests, three interactions remained significant: sex in the SPMSQ period (FDR-adjusted P = 0.036) and age and comorbidity in the AD8 period (both FDR-adjusted P = 0.004); the remaining interactions were not significant after adjustment (S3 Table).”

**Discussion, Limitations (p. 30–31, lines 454–463):**

“In addition, formal testing indicated some departure from the proportional hazards assumption for marital status, concentrated in the widowed and divorced/separated groups (whose associations were null); the never-married association central to our findings showed little or no such departure, and the reported hazard ratios should be interpreted as effects averaged over the follow-up period. Finally, because linked mortality data were incomplete, death could not be modeled as a competing event, and differential mortality by marital status could introduce informative censoring; our estimates therefore represent cause-specific hazards, and the small proportion of participants excluded for incomplete data (<2%) is unlikely to have biased the results.”

**COMMENT 7:**

The discussion is overly speculative.

**RESPONSE TO COMMENT 7:**

We thank the reviewer for this feedback and agree that our original Discussion devoted disproportionate space to elaborating and defending specific mechanistic explanations for the observed reversal in never-married status risk, even though we ourselves had already acknowledged that the reversal might be entirely attributable to measurement heterogeneity rather than a genuine effect. We have substantially condensed the Discussion as follows. First, we reduced the “Potential Mechanisms” section from three paragraphs enumerating four distinct hypothesized pathways (lifelong independence, decision-making autonomy, varied social-network stimulation, and differential digital-technology adoption) to a single, shorter paragraph that names only the broadest plausible category of explanation and explicitly declines to pursue mechanism-specific speculation, particularly removing the digital-technology hypothesis, which our own text had already characterized as “entirely speculative” given the absence of any technology-use data in our cohort. Second, we revised the sex-, age-, and education-stratified discussion to report subgroup patterns descriptively, explicitly stating that we do not attempt to attribute them to specific mechanisms (e.g., shifting sex-role expectations or age-specific adaptive capacity) given the measurement heterogeneity already discussed elsewhere in the Discussion, and we now highlight only the one subgroup interaction that remained significant after correction for multiple comparisons. Third, we revised the Clinical and Public Health Implications paragraph to remove specific policy recommendations (e.g., digital-inclusion programs) that had been tied to the now-removed speculative mechanisms, replacing them with a more conservative statement that explicit replication is needed before any practice or policy implications can be drawn. We believe these revisions bring the tone and scope of the Discussion into better proportion with the strength of evidence the study can actually support.

**Revised text:**

**Discussion, Potential Mechanisms (p. 27, lines 364–379):**

“The hypothetical mechanisms outlined below assume that the observed reversal reflects, at least in part, a genuine temporal change rather than measurement heterogeneity between the SPMSQ and AD8 periods (see Limitations); we offer them only as illustrative possibilities for future testing, not as confirmed explanations, and we deliberately limit ourselves to the broadest plausible categories rather than an exhaustive list of pathways. During the earlier period, married individuals may have benefited from greater social support and health-behavior monitoring through spousal interaction, consistent with the stress-buffering hypothesis [13, 31, 32]; never-married individuals may have faced relative disadvantage in these domains. Any protective association observed for never-married status in the later period would be broadly consistent with a general hypothesis of lifelong independence fostering adaptive self-reliance and social-network management [33-35] , though our study design cannot test this, nor distinguish it from the non-mechanistic, measurement-based explanations discussed above. We did not collect data on social network characteristics, decision-making autonomy, or technology use, and therefore do not pursue mechanism-specific speculation further beyond this general hypothesis; detailed assessment of these constructs is an important direction for future mixed-methods research (see Limitations).”

**Discussion, Sex and Age Interactions (p. 27–28, lines 381–390):**

“Sex-stratified analyses showed elevated MCI risk among never-married men (HR=1.89, 95% CI: 1.41-2.54) and divorced women (HR=1.78, 95% CI: 1.20-2.65) during 2005-2011, broadly consistent with literature on sex differences in spousal dependence and the economic impact of marital dissolution [13, 31, 49, 51]; both patterns reversed or attenuated during 2012-2016. The age interaction during the AD8 period—a protective association among younger-old adults (65-79 years: HR=0.42) that was absent among the oldest-old—was the only subgroup interaction to remain significant after correction for multiple comparisons (S3 Table). Given the measurement heterogeneity discussed above, we do not attempt to attribute these subgroup patterns to specific mechanisms (e.g., shifting sex-role expectations or age-specific adaptive capacity); we report them descriptively as patterns warranting replication.”

**Discussion, education-stratified paragraph (p. 28, lines 390–396):**

“During 2005-2011, adverse associations of non-married status were concentrated among lower-educated individuals, broadly consistent with cognitive reserve theory [53, 54]; this education-stratified pattern was not observed during 2012-2016, when protective associations for never-married status appeared across educational strata. As with the sex and age patterns above, we report this descriptively rather than attributing it to a specific mechanism, given the measurement heterogeneity already discussed.”

**Discussion, Clinical and Public Health Implications (p. 28, lines 398–406):**

“Given the uncertainty surrounding the interpretation of our findings, we offer only tentative implications. Clinicians should be cautious about assuming marriage universally confers cognitive protection, and our findings do not, on their own, support specific intervention targets for never-married older adults; replication using consistent measurement approaches across periods is needed before practice or policy recommendations can be drawn. More broadly, our findings underscore the value of longitudinal monitoring of social determinants of cognitive health using consistent assessment methods, so that genuine temporal change can be distinguished from measurement artifact when evaluating how such associations evolve with societal change.”

**REVIEWER 2 COMMENTS**

While the study utilizes an impressive longitudinal cohort (n=103,863) and addresses a critical gap in measurement heterogeneity, several methodological concerns must be addressed to ensure the robustness of your findings. Below are the formal recommendations for revision:

**INTRODUCTION**

**COMMENT 1:**

The Introduction should explicitly address the potential for reverse causality, a well-known challenge in social epidemiology. It is plausible that incipient cognitive decline, even in subclinical stages, may influence an individual’s marital status or their likelihood of maintaining a long-term marriage. Highlighting this conceptual limitation early in the paper is essential for framing the study’s theoretical boundaries before presenting the methodology.

**RESPONSE TO COMMENT 1:**

We thank the reviewer for this valuable suggestion and agree that this conceptual limitation should be framed explicitly and early in the manuscript, before the Methods. We have added a new paragraph to the Introduction that explicitly names reverse causality as a well-recognized methodological challenge in this literature, drawing on prior work (Håkansson et al., BMJ 2009) that specifically identified reverse causation—subclinical cognitive decline preceding and potentially influencing marital transitions—as a major source of bias in studies of marital status and cognitive outcomes, as well as a study using lagged marital status to address this concern in dementia research (Zhang et al., 2021). The new paragraph explains that incipient, undetected cognitive decline may influence an individual’s likelihood of marrying, remaining married, or experiencing marital dissolution, and explicitly previews that our analytic strategy (lag analyses) is intended to partially address this concern, which is described further in the Methods and Limitations. We believe this addition appropriately frames the theoretical boundaries of the study before the Methods are presented.

**Revised text:**

**Introduction, new paragraph (p. 5, lines 78–91):**

“A related conceptual challenge in this literature is the possibility of reverse causality [15, 19]. Rather than marital status causally shaping cognitive trajectories, incipient cognitive decline—even at a subclinical stage, before it would be detected on a screening instrument—may itself influence an individual’s likelihood of marrying, remaining married, or experiencing marital dissolution. Individuals with early, undetected cognitive changes may be less likely to initiate or sustain a long-term marriage, more likely to experience divorce or separation as a result of relationship strain, or, conversely, more likely to remain married if a spouse compensates for emerging functional difficulties in ways that delay formal detection. This possibility is particularly salient in observational cohorts with infrequent assessment intervals, where subclinical decline may precede the first recorded cognitive assessment by several years. Although our analytic strategy includes lag analyses intended to mitigate this concern (see Methods), the baseline ascertainment of marital status cannot fully rule out reverse causal pathways, and this conceptual limitation should be borne in mind when interpreting the associations reported below.”

**Reference list addition (#19):**

“Håkansson K, Rovio S, Helkala EL, Vilska AR, Winblad B, Soininen H, et al. Association between mid-life marital status and cognitive function in later life: population based cohort study. Bmj. 2009;339:b2462. Epub 20090702. doi: 10.1136/bmj.b2462. PubMed PMID: 19574312; PubMed Central PMCID: PMCPMC2714683.”

**MATERIALS AND METHODS**

**COMMENT 2 and 3:**

- The cohort is derived from adults participating in voluntary government-sponsored health examinations. The authors should explicitly address the potential for selection bias, as this population is likely more health-conscious and possesses higher health literacy than the general elderly population in Taiwan.
- The patient selection process, as detailed in Fig 1 and the Methods section, reveals a significant loss of data that may compromise the representativeness of the findings. Specifically, the study excluded 44,254 individuals from the SPMSQ period and 32,700 from the AD8 period because they had a “single examination”. This equates to approximately 42% of the initial cohort. In geriatric epidemiology, it is well-documented that participants who fail to return for follow-up assessments are typically frailer, more ill, or face more precarious socioeconomic conditions than those who remain in the study. By excluding nearly half of the original population, the results are highly susceptible to a “healthy volunteer” or “healthy survivor” bias, which may lead to an underestimation of the true associations between marital status and cognitive decline. To address this limitation, the authors should provide a comparative analysis of baseline characteristics (e.g., age, comorbidities, education, and marital status) between the included analytic sample and the individuals excluded due to having only a single examination. This comparison, which could be included in the supplementary material, is essential to evaluate the direction and magnitude of the potential selection bias.

**RESPONSE TO COMMENT 2 and 3:**

We thank the reviewer for this important point and for the opportunity to evaluate the potential selection bias directly. We have added **S4 Table**, which compares the baseline characteristics of the analytic cohort with those of the single-examination participants excluded from the longitudinal analysis (n = 44,254 in the SPMSQ period and n = 32,700 in the AD8 period), separately for each period, using standardized mean differences (SMDs); an absolute SMD below 0.10 indicates a negligible between-group difference (Austin, 2009).

Across both periods, the two groups were similar on most characteristics, including the entire clinical comorbidity profile—hypertension, diabetes, dyslipidemia, heart disease, stroke, and psychiatric history (all |SMD| < 0.10)—as well as sex, low-income status, BMI category, and alcohol use. Importantly, excluded participants were not more burdened by comorbidity (prevalences were comparable or marginally lower), which does not support the premise that they constituted a frailer or more severely ill subgroup. The differences that were present were concentrated in social and educational characteristics: excluded participants were less educated (SMD = 0.28 [SPMSQ] and 0.16 [AD8]) and more often never-married (0.26 and 0.23) in both periods; in addition, they were slightly more likely to live alone (0.11) and to smoke (0.12) in the SPMSQ period, and were somewhat older (0.19) and more likely to report psychological distress (0.19) in the AD8 period. Age differed negligibly in the SPMSQ period (73.5 vs. 74.0 years; SMD = 0.07).

All of the characteristics that differed (education, marital status, age, living arrangement, smoking, psychological distress) are included as covariates in the multivariable models, so their imbalance is accounted for in the adjusted estimates. Moreover, the never-married association central to our findings was robust in the sensitivity analyses already reported, including the time-varying marital-status model (S1 Table) and the ≥2-year lag analysis (Table 5), which would not be expected were the association an artifact of differential selection. We nonetheless agree that, as with any cohort drawn from voluntary government-sponsored health examinations, the analytic sample is likely healthier and more health-literate than the general older Taiwanese population, and residual selection toward a higher-functioning sample cannot be excluded; such selection would, if anything, bias the observed associations toward the null. We have added these points to the Limitations and now reference S4 Table.

**Revised text:**

**Discussion, Limitations (p. 31, lines 463–470):**

“Beyond this, because the analytic cohort required at least two examinations, participants with a single examination were excluded; a comparison of baseline characteristics between the analytic cohort and these participants (S4 Table) showed comparable clinical comorbidity, sex, income, and body-mass distributions (all standardized mean differences < 0.10), with excluded participants being less educated and more often never-married and differing modestly on a few additional characteristics, all of which are adjusted for in our models. Residual selection toward a higher-functioning sample nonetheless cannot be excluded and would, if anything, bias the observed associations toward the null.”

**Reference:**

Austin, P. C. (2009). Balance diagnostics for comparing the distribution of baseline covariates between treatment groups in propensity-score matched samples. *Statistics in Medicine, 28*(25), 3083–3107.

**COMMENT 4:**

The study treats marital status as a “fixed characteristic” based on baseline data. Given that the mean follow-up duration reached 3.7 years for the SPMSQ period and 2.8 years for the AD8 period, it is highly probable that some participants experienced marital transitions (e.g., becoming widowed or divorced) during the course of the study. While other health variables were time-updated, keeping marital status fixed may introduce misclassification bias. I recommend treating marital status as a time-dependent covariate to align with the rest of the model.

**RESPONSE TO COMMENT 4:**

We thank the reviewer for this suggestion. Marital status was ascertained at examination, and within-person updates were available only in the later study period (2012–2016); in the earlier period (2005–2011) marital status was recorded as a fixed baseline value without within-person variation. For the SPMSQ period we therefore used baseline marital status, as the source data do not support a time-varying specification for this period. For the AD8 period, where within-person updates were available, we re-fitted the model treating marital status as a time-varying covariate within the same counting-process framework used for the other time-updated covariates. The estimates were materially unchanged: never-married status remained significantly protective (HR = 0.63, 95% CI 0.49–0.81, vs. baseline HR = 0.60, 0.46–0.77), and the divorced and widowed contrasts remained non-significant (S1 Table). These results indicate that exposure misclassification from marital transitions does not account for our findings. We have clarified the Methods accordingly and added a limitation noting that the two periods were derived from data sources with differing marital-status ascertainment.

**COMMENT 5:**

While the study aims to examine “incident mild cognitive impairment (MCI),” it is important to address a critical terminological distinction regarding the instruments employed. Neither the SPMSQ nor the AD8 questionnaire constitutes a formal clinical diagnosis of MCI. By design, both are screening instruments rather than diagnostic tools. I recommend that the authors avoid the definitive term “incident MCI” throughout the text. Instead, more methodologically accurate expressions such as “screen-positive cognitive impairment” or “probable cognitive impairment” should be used to reflect the limitations of using screening thresholds (SPMSQ ≥3 and AD8 ≥2) to define the study outcomes.

**RESPONSE TO COMMENT 5:**

We thank the reviewer for raising this important terminological distinction. We agree that the SPMSQ and AD8 are screening instruments rather than clinical diagnostic tools, and that the term “incident MCI” could overstate diagnostic certainty. We have revised the manuscript to explicitly reframe our outcome as “screen-positive cognitive impairment,” while retaining “MCI” as a defined abbreviation for this screen-positive status (rather than for a clinical diagnosis), for brevity and consistency with prior literature using these instruments. Specifically, we revised the main title and abstract to read “incident screen-positive cognitive impairment (MCI)”; added an explicit clarifying statement in the Methods (Measures of cognitive function) that both instruments are validated screening tools rather than diagnostic instruments, and that the resulting classification reflects screen-positive status based on a standardized cutoff rather than a clinical diagnosis of mild cognitive impairment or dementia; and updated the titles of Tables 3, 4, and 5 to read “incident screen-positive cognitive impairment (MCI).” Because the abbreviation MCI is now formally defined as “screen-positive cognitive impairment” at first use, its subsequent use throughout the Results, Discussion, and table bodies no longer implies a clinical diagnosis.

**Revised text:**

**Title (p. 1, lines 1–3):**

“Marital status and incident screen-positive cognitive impairment in a Taiwanese longitudinal cohort (2005–2016): Associations vary by cognitive screening instrument”

**Short title:**

*Note: a search of the manuscript file did not locate a separate “Short title” line in the document body. PLOS ONE collects the short/running title as a metadata field during submission in Editorial Manager rather than as text inside the manuscript itself. Please confirm the short title text entered in Editorial Manager directly; if it still needs to be updated to reflect the “screen-positive cognitive impairment” terminology, it should be revised there rather than at a page/line citation in the manuscript file.*

**Abstract, Background (p. 2, lines 21–24):**

“This study examined whether associations between marital status and incident screen-positive cognitive impairment (MCI) differ by screening instrument and population subgroups in a large longitudinal cohort of community-dwelling older adults in Taiwan.”

**Methods, Measures of cognitive function (p. 9, lines 131–136):**

“In this study, screen-positive cognitive impairment (hereafter referred to as MCI for brevity and consistency with prior literature using these instruments) was ascertained using the SPMSQ and AD8 questionnaires. As both the SPMSQ and AD8 are validated screening tools rather than clinical diagnostic instruments, the resulting classification reflects screen-positive status based on a standardized cutoff rather than a clinical diagnosis of mild cognitive impairment or dementia.”

**Table 3 title (p. 20, lines 263–264):**

“Table 3. Cox proportional hazards models of marital status predicting incident screen-positive cognitive impairment (MCI)”

**Table 4 title (p. 22, line 283):**

“Table 4. Subgroup Cox models of marital status and incident screen-positive cognitive impairment, stratified by sex, age, and education”

**Table 5 title (p. 24, line 301):**

“Table 5. Sensitivity analyses of Cox proportional hazards models of marital status predicting incident screen-positive cognitive impairment (MCI)”

**COMMENT 6:**

There is a significant discrepancy between the “Covariates” section of the manuscript and the variables listed in the final analytical models (Tables 3–5). In the primary description of health-related covariates, the authors mention hypertension, diabetes, hyperlipidemia, heart disease, psychotropic or insomnia medication use, smoking, and alcohol. However, the notes for Table 3, Table 4, and Table 5, as well as a later subsection of the statistical analysis, include additional variables such as stroke, psychiatric history, and psychological distress. None of these additional variables are formally defined. The manuscript does not specify whether they were assessed via self-report, clinical diagnosis, or ICD codes from the database. The “Covariates” section should be updated to include an exhaustive and precise list of every variable included in the final models. Furthermore, the authors must explicitly define how stroke and psychiatric history were measured to allow for proper replication and validation of the results.

**RESPONSE TO COMMENT 6:**

We thank the reviewer for this careful reading and for the opportunity to make the covariate specification fully transparent. The reviewer is correct that the “Covariates” subsection was not fully aligned with the variables listed in the final models and that the ascertainment of several variables was not stated. We have revised the “Covariates” subsection to (i) list exhaustively every variable entered into the fully adjusted model and (ii) state precisely how each was measured. We confirm that all covariates were obtained from the self-administered health-examination questionnaire or from administrative linkage; none were derived from clinical diagnoses or ICD-coded records.

**Definition and ascertainment of the covariates.** The chronic-disease covariates (hypertension, diabetes, hyperlipidemia, heart disease, and stroke) were ascertained from the questionnaire on the basis of self-reported current medication use together with its reported indication; each was coded as present (yes/no) when a participant reported taking medication for that condition (for stroke, an indication of stroke or cerebrovascular disease). “Psychiatric history” denotes the same variable that the original “Covariates” text had labeled “psychotropic or insomnia medication use”; this inconsistent labeling was the source of the discrepancy the reviewer identified. It was defined as self-reported use of medication for a psychiatric or psychological indication (e.g., depression, anxiety, or other psychiatric or psychosomatic conditions) or of sedative-hypnotic medication for insomnia. “Psychological distress” was measured with the 5-item Brief Symptom Rating Scale (BSRS-5), a validated screening instrument embedded in the examination questionnaire; the total score (range 0–20) was dichotomized at the established cutoff of ≥6 to indicate the presence of psychological distress.

**Exhaustive covariate list and harmonized terminology.** To remove the inconsistency, we now use the single term “psychiatric history” throughout the “Covariates” text, the statistical-analysis subsection, the baseline-characteristics tables (Tables 1 and 2), and the footnotes to Tables 3–5. Stroke had been adjusted for in the models (as shown in those footnotes) but was inadvertently omitted from the “Covariates” text; it is now defined and listed. The fully adjusted model (Model 2) included age, sex, education, low-income household status, living arrangement, body-mass-index category, hypertension, diabetes, hyperlipidemia, heart disease, stroke, psychiatric history, psychological distress, current smoking, and alcohol consumption.

**Revised text:**

**Methods, Covariates (p. 11–12, lines 170–185):**

“The chronic-disease covariates—hypertension, diabetes, hyperlipidemia, heart disease, and stroke—were ascertained from the self-administered questionnaire on the basis of self-reported current medication use and its reported indication. … Psychiatric history was defined in the same manner, as self-reported use of medication for a psychiatric or psychological indication … or of sedative-hypnotic medication for insomnia. Psychological distress was measured using the 5-item Brief Symptom Rating Scale (BSRS-5) … dichotomized at the established cutoff of ≥6 … Thus, the fully adjusted model (Model 2) included age, sex, education, low-income household status, living arrangement, BMI category, hypertension, diabetes, hyperlipidemia, heart disease, stroke, psychiatric history, psychological distress, current smoking, and alcohol consumption. None of these covariates were derived from clinical diagnoses or ICD-coded records…”

*(Note: the four passages joined by “…” above are each verbatim, contiguous text in the manuscript, but they are not contiguous with one another — they are separated by other sentences within the same Methods/Covariates subsection at p. 11–12, lines 170–185. A reviewer searching for any individual segment between the ellipses will find it verbatim.)*

**Methods, Statistical analyses (p. 12, lines 194–198):**

“Model 2 adjusted for baseline covariates (age, sex, education, living arrangement, low-income status) and time-dependent covariates reassessed at each follow-up (BMI, histories of hypertension, diabetes, dyslipidemia, heart disease, stroke, psychiatric history, psychological distress, smoking, alcohol consumption).”

**COMMENT 7:**

While the models adjusted for “psychotropic or insomnia medication use” and “psychological distress”, clinical depression is a potent confounder for both social isolation and cognitive test performance (particularly on the SPMSQ). If available, diagnostic data for depression should be included to minimize residual confounding.

**RESPONSE TO COMMENT 7:**

We thank the reviewer for raising this important point, and we agree that clinical depression is a potential confounder of both social connectedness and cognitive test performance. The health-examination database used in this study does not contain a formal clinical or ICD-coded diagnosis of depression, so a re-analysis incorporating a diagnostic depression variable is not feasible. However, two questionnaire-based indicators that capture depressive illness and depressive symptomatology were already included in all adjusted models: (i) psychiatric history, based on self-reported use of medication for psychiatric or psychological indications (including antidepressant and anxiolytic medication), and (ii) psychological distress, measured by the BSRS-5, a screening scale whose items index depressive and anxiety symptoms. These covariates partially mitigate the concern, although we acknowledge that they are not equivalent to a formal diagnosis. We have added an explicit statement of this limitation to the Discussion.

**Revised text:**

**Discussion, Limitations (p. 31, lines 470–474):**

“Although the models adjusted for self-reported psychiatric medication use and for psychological distress measured by the BSRS-5, the database did not include a formal clinical or ICD-coded diagnosis of depression; residual confounding by undiagnosed or untreated depression therefore cannot be entirely excluded.”

**RESULTS**

**COMMENT 8:**

The nature of the AD8 represents a major threat to the validity of the results for the “never-married” group. The AD8 relies on an informant to detect functional change. However, the data show that never-married individuals are far more likely to live alone (20%) compared to married individuals (0.9%). If a never-married participant lives alone and lacks a daily informant, the study likely relied on self-reporting, which is known to underestimate cognitive decline in early stages. The “protective” effect observed for never-married individuals in the AD8 period may simply be a misclassification error due to a lack of external observation.

**RESPONSE TO COMMENT 8:**

We thank the reviewer for raising this important point regarding a potential informant-related ascertainment bias. We agree that this is a serious threat to the validity of the protective association observed for never-married status during the AD8 period, and we have addressed it explicitly in three places in the manuscript. First, in the Methods, we disclose that the database did not record whether each AD8 assessment was completed by the participant or by a caregiver/informant, which precludes direct stratification by respondent type and therefore direct testing of the reviewer’s hypothesis. Second, in the Discussion, we explicitly lay out the mechanism the reviewer describes: because married individuals more often have a co-residing spouse available to detect subtle functional decline, whereas never-married individuals more often rely on self-report, and because self-report is known to under-detect early cognitive decline relative to informant report, this differential ascertainment — together with the anosognosia (impaired insight) that is common in early cognitive impairment — is the more likely explanation for the lower hazard observed among never-married participants during the AD8 period, rather than a true protective effect. Third, in the Limitations, we state explicitly that, although our models adjusted for living arrangement (alone vs. with others), which partially, but not fully, captures the availability of a co-residing observer, the database did not allow direct testing of respondent type, and that this informant-related ascertainment bias is the more likely explanation for the lower hazard observed among never-married participants during the AD8 period. We note that the living-alone percentages cited by the reviewer (20.0% among never-married vs. 0.9% among married participants during the AD8 period) match those reported in Table 2, and that this informant-related bias is considered alongside, and is not fully separable from, the broader measurement heterogeneity between the SPMSQ and AD8 periods discussed elsewhere in the manuscript.

**Revised text:**

**Methods, Measures of cognitive function (p. 10, lines 148–152):**

“This assessment tool offers flexibility in administration, allowing completion through either caregiver observation or individual self-evaluation; however, the database did not record which respondent type (self vs. caregiver/informant) was used for each assessment, precluding direct stratification by informant type.”

**Discussion (p. 26, lines 338–348):**

*(This sentence was further sharpened in response to Comment 12 below; the text below is the final version, identical to the text reproduced under Comments 2 and 12.)*

“In particular, because the AD8 can be completed by either a co-residing informant or the participant, married individuals—who more often have a spouse available to observe subtle functional decline—may be more likely to be flagged as having cognitive impairment than never-married individuals, who more often rely on self-report; self-report is known to under-detect early cognitive decline relative to informant report, in part because of anosognosia (impaired insight into one’s own cognitive deficits) in the early stages of impairment. Given that never-married individuals in our cohort were markedly more likely to live alone (20.0% vs. 0.9% among married participants during the AD8 period), we consider this differential ascertainment by respondent type, rather than a true protective effect of never-married status, the more likely explanation for the reduced hazard observed during the AD8 period.”

**Limitations (p. 29, lines 414–426):**

*(This sentence was further strengthened in response to Comment 12 below; the text below is the final version, identical to the text reproduced under Comments 2 and 12.)*

“The SPMSQ and AD8 assess different cognitive domains and rely on different informant sources, which may differentially capture cognitive changes across marital status groups [28, 30]. Although our models adjusted for living arrangement (alone vs. with others), which partially captures the availability of a co-residing observer, the database did not record whether each AD8 assessment was completed by the participant or by a caregiver/informant, precluding direct testing of this hypothesis. Because married individuals are more likely to have a co-residing spousal informant who can detect subtle functional decline, whereas never-married individuals more often rely on self-report—a method prone to under-detection of early decline due to anosognosia—differential detection by respondent type, rather than a true protective effect, is the more likely explanation for the lower hazard observed among never-married participants during the AD8 period; this informant-related ascertainment bias should be considered alongside, and is not fully separable from, the broader measurement heterogeneity discussed above.”

**COMMENT 9:**

The mean follow-up for the SPMSQ period was 3.7 years, whereas the AD8 period was only 2.8 years. Cognitive decline is time-dependent. A shorter follow-up in the second period might only capture rapid-onset cases, while the first period captures a slower progression. While Cox models adjust for time-at-risk, this nearly one-year difference in observation density adds significant noise to the comparison of HRs between the two historical periods.

**RESPONSE TO COMMENT 9:**

We thank the reviewer for this important methodological point. We agree that the nearly one-year difference in mean follow-up between the SPMSQ period (3.7 years) and the AD8 period (2.8 years) is a potential source of imprecision in comparing hazard ratios across periods, beyond the simple confounding of follow-up duration with calendar period already noted elsewhere in the manuscript. We have therefore added explicit discussion of this mechanism in two places. In the Discussion, we now state that the shorter mean follow-up in the AD8 period may have disproportionately captured more rapid-onset cases while under-ascertaining the slower, more gradual cognitive decline that a longer observation window would detect, and that this difference in observation density adds a further source of imprecision to any direct between-period comparison of hazard ratios. In the Limitations, we similarly state that the two periods likely differ in observation density and in their relative sensitivity to rapid-onset versus slowly progressive cognitive decline, which adds further noise to any direct comparison of hazard ratios beyond what Cox models—which adjust for time-at-risk but not for this differential case-mix—can fully account for. We note that our existing sensitivity analyses stratified by follow-up duration (Table 5) and the two-year lag analysis remain informative as robustness checks, but, as now stated explicitly, cannot fully resolve this source of between-period imprecision.

**Revised text:**

**Discussion (p. 25, lines 325–331):**

“In addition, because cognitive decline is time-dependent, the shorter mean follow-up in the AD8 period (2.8 years) relative to the SPMSQ period (3.7 years) may have disproportionately captured more rapid-onset cases while under-ascertaining the slower, more gradual cognitive decline that a longer observation window would detect; this near one-year difference in observation density adds a further source of imprecision—beyond simple confounding by calendar period—to any direct comparison of hazard ratios across the two periods.”

**Limitations (p. 30, lines 440–446):**

“Fourth, relatively short follow-up periods (median 3.2-3.8 years) may not capture the full spectrum of marital status effects on cognitive aging; moreover, because the AD8 period’s mean follow-up (2.8 years) was nearly one year shorter than that of the SPMSQ period (3.7 years), the two periods likely differ in observation density and in their relative sensitivity to rapid-onset versus slowly progressive cognitive decline, which adds further noise to any direct comparison of hazard ratios between periods beyond what Cox models, which adjust for time-at-risk, can fully account for.”

**COMMENT 10:**

The Results section frequently conflates objective data description with interpretation and discussion. In several passages, the authors do not merely report the findings but offer premature interpretations. For instance, the text states that “risk patterns reversed” and explicitly claims that “effect modification by sex and age was observed”. Such interpretive language and the labeling of statistical trends should be reserved for the Discussion section. The Results section should maintain a neutral tone, focusing strictly on the reporting of data, including Hazard Ratios, 95% Confidence Intervals, and interaction p-values, without assigning clinical or theoretical meaning at this stage.

**RESPONSE TO COMMENT 10:**

We thank the reviewer for this important observation. We agree that the Results section had, in several places, used interpretive or labeling language (e.g., “reversed,” “became protective,” “demonstrated significant effect modification,” “excluded reverse causation”) that assigns clinical or theoretical meaning to the data rather than simply reporting it, and that such interpretation is more appropriately reserved for the Discussion. We have systematically revised the Results section to report only hazard ratios, 95% confidence intervals, and interaction p-values in neutral, descriptive language, removing all directional/causal labels and evaluative verbs (e.g., “reversed,” “protective,” “demonstrated,” “confirmed,” “excluded”). The corresponding interpretation of these patterns—including the possibility that they reflect measurement heterogeneity rather than a genuine temporal change—is already presented in the Discussion and Limitations, as described in our responses to other comments.

**Revised text:**

**Results, AD8 period primary finding (p. 19, lines 259–261):**

“In the AD8 period, the HR for never-married status was 0.60 (95% CI: 0.46–0.77), while divorced (HR=1.03, 95% CI: 0.83–1.27) and widowed (HR=1.04, 95% CI: 0.93–1.17) participants showed non-significant associations.”

**Results, sex-stratified findings (p. 21, lines 270–273):**

“Table 4 presents stratified Cox regression analyses. During the SPMSQ period, the HR for never-married status was 1.89 (95% CI: 1.41–2.54) in males and the HR for divorced status was 1.78 (95% CI: 1.20–2.65) in females. During the AD8 period, the HR for never-married status was 0.66 in males and 0.53 in females.”

**Results, age-stratified findings (p. 21, lines 273–277):**

“In the SPMSQ period, the HR for never-married status was 1.66 (95% CI: 1.19–2.30) among participants aged 65–79 years and was non-significant among those ≥80 years. In the AD8 period, the HR for never-married status was 0.42 (95% CI: 0.29–0.61) among participants aged 65–79 years and was non-significant among those ≥80 years.”

**Results, education-stratified findings (p. 21, lines 277–281):**

“In the SPMSQ period, the HRs for divorced and never-married status were 1.61 and 1.48, respectively, among participants with <10 years of education, and were non-significant among those with ≥10 years of education. In the AD8 period, the HR for never-married status was 0.69 among participants with <10 years of education and 0.50 among those with ≥10 years of education.”

**Results, subgroup interaction analyses (p. 23, lines 287–291):**

“Subgroup analyses examined potential effect modification across periods (Fig 2). During SPMSQ assessment, the marital status × sex interaction was significant (P=0.008). During AD8 assessment, the marital status × age interaction (P<0.001) and marital status × chronic disease interaction (P=0.001) were significant. The interactions for education, BMI, living arrangement, and psychological distress were not significant.”

**Results, sensitivity analyses summary (p. 23, lines 295–296):**

“Sensitivity analyses stratified by follow-up duration and lag time are presented in Table 5.”

**Results, two-year lag analysis (p. 23, lines 298–299):**

“The two-year lag analysis yielded an HR of 0.57 (95% CI: 0.44–0.75) for never-married status.”

**DISCUSSION**

**COMMENT 11:**

The discussion attributes the risk reversal to “societal transformations” and the “digital revolution” between the 2005–2011 and 2012–2016 periods. However, this shift coincides exactly with the transition from the SPMSQ to the AD8. It is methodologically risky to suggest that social norms changed so radically in a single year (the transition point). The authors should prioritize the measurement artifact explanation over the social change hypothesis. It must be stated that the “temporal authenticity” claimed is highly questionable given the lack of an overlap period where both instruments were used simultaneously to calibrate results.

**RESPONSE TO COMMENT 11:**

We thank the reviewer for this important methodological critique. We agree that our original framing, which attributed the observed reversal to “societal transformations” and the “digital revolution,” overstated the case for a genuine temporal change and did not adequately weigh the more parsimonious measurement-artifact explanation. We have removed this language and revised the Discussion to state explicitly that, because the transition from SPMSQ to AD8 coincides exactly with the boundary between the two calendar periods, and because the dataset contains no interval in which both instruments were administered concurrently to allow direct calibration of one against the other, we cannot empirically distinguish a genuine secular change in the marital status–cognition relationship from an artifact of the instrument transition. We now state explicitly that, given this lack of overlap-period calibration, the measurement-artifact explanation is regarded as the more parsimonious interpretation and is prioritized over a social-change account in the conclusions we draw. We have correspondingly reframed the “Potential Mechanisms” section so that any substantive mechanisms (e.g., adaptive self-reliance) are presented only as illustrative possibilities conditional on a genuine temporal change, rather than as our preferred explanation. We have also added a parallel statement to the Limitations, reiterating that the absence of an overlap period precludes direct calibration between the two instruments and further limits our ability to attribute the observed reversal to genuine temporal change rather than to the instrument transition itself.

**Revised text:**

**Discussion (p. 26–27, lines 355–362):**

“Indeed, because the transition from SPMSQ to AD8 coincides exactly with the boundary between the two calendar periods, and because the dataset does not include any interval in which both instruments were administered concurrently to allow direct calibration of one against the other, we cannot empirically distinguish a genuine secular change in the marital status–cognition relationship from an artifact of the instrument transition; given this lack of overlap-period calibration, the measurement-artifact explanation should be regarded as the more parsimonious interpretation and is prioritized over a social-change account in the conclusions we draw.”

**Limitations (p. 30, lines 434–437):**

“Because the two periods do not include an interval in which both instruments were administered concurrently, no direct calibration between SPMSQ and AD8 scores is possible, further limiting our ability to attribute the observed reversal to genuine temporal change rather than to the instrument transition itself.”

**COMMENT 12:**

The authors suggest that never-married individuals might possess “subtle cognitive advantages” or “greater adaptive capacity” in the second period. They overlook the fact that the AD8 is an informant-based instrument. The data show that never-married individuals are significantly more likely to live alone compared to married individuals. In the absence of a co-residing spouse to observe daily functional decline, the AD8 likely relied on self-reporting, which is prone to anosognosia (lack of insight) in early stages of impairment. The discussion must explicitly recognize that the “reduced risk” in this group is likely a detection bias resulting from the lack of a reliable informant.

**RESPONSE TO COMMENT 12:**

We thank the reviewer for this important critique. We agree that our original framing, which suggested that never-married individuals might possess “subtle cognitive advantages” associated with “adaptive social behaviors” during the AD8 period, overlooked the informant-dependent nature of the AD8 and did not adequately credit the more parsimonious detection-bias explanation. We have revised the Discussion to remove this language and to state explicitly that, because never-married individuals in our cohort were markedly more likely to live alone than married individuals (20.0% vs. 0.9% during the AD8 period) and therefore more likely to rely on self-report rather than informant report, and because self-report is known to under-detect early cognitive decline in part due to anosognosia, this differential ascertainment by respondent type is the more likely explanation for the reduced hazard observed among never-married participants, rather than any genuine cognitive advantage. We have correspondingly removed the sentence proposing that the AD8 “may better capture subtle cognitive advantages associated with adaptive social behaviors,” and replaced it with a statement attributing the apparent reversal to this instrument-driven detection bias. We have made the parallel statement in the Limitations section similarly explicit, upgrading the language from “remains a plausible explanation” to “is the more likely explanation.”

**Revised text:**

**Discussion (p. 26, lines 341–348):**

“…impairment than never-married individuals, who more often rely on self-report; self-report is known to under-detect early cognitive decline relative to informant report, in part because of anosognosia (impaired insight into one’s own cognitive deficits) in the early stages of impairment. Given that never-married individuals in our cohort were markedly more likely to live alone (20.0% vs. 0.9% among married participants during the AD8 period), we consider this differential ascertainment by respondent type, rather than a true protective effect of never-married status, the more likely explanation for the reduced hazard observed during the AD8 period.”

**Discussion (p. 26, lines 348–352):**

“The SPMSQ’s focus on crystallized cognitive abilities may have been more sensitive to traditional risk factors, while the AD8’s reliance on informant report makes it more susceptible to the differential ascertainment bias described above [28, 29]; we therefore consider this instrument-driven detection bias, rather than any genuine cognitive advantage among never-married individuals, the more likely driver of the apparent reversal.”

**Limitations (p. 29, lines 419–426):**

“Because married individuals are more likely to have a co-residing spousal informant who can detect subtle functional decline, whereas never-married individuals more often rely on self-report—a method prone to under-detection of early decline due to anosognosia—differential detection by respondent type, rather than a true protective effect, is the more likely explanation for the lower hazard observed among never-married participants during the AD8 period; this informant-related ascertainment bias should be considered alongside, and is not fully separable from, the broader measurement heterogeneity discussed above.”
