# Supplementary material for: Marital status and incident screen-positive cognitive impairment in a Taiwanese longitudinal cohort (2005–2016): Associations vary by cognitive screening instrument
Source: PLoS One. 2026 Oct 5;21(10):e0359778. doi: 10.1371/journal.pone.0359778 (PMC13637897; doi:10.1371/journal.pone.0359778)
Supplement: Peer review history

# Peer review: Decision Letter 0

**DOI:** 10.1371/journal.pone.0359778.r001

**Authors:** Muhammad Shakaib

11 Jun 2026

Dear Dr. Zhou,

Thank you for submitting your manuscript to PLOS ONE. After careful consideration, we feel that it has merit but does not fully meet PLOS ONE’s publication criteria as it currently stands. Therefore, we invite you to submit a revised version of the manuscript that addresses the points raised during the review process.

plosone@plos.org. When you're ready to submit your revision, log on to https://www.editorialmanager.com/pone/ and select the 'Submissions Needing Revision' folder to locate your manuscript file.

- A letter that responds to each point raised by the academic editor and reviewer(s). You should upload this letter as a separate file labeled 'Response to Reviewers'.
- A marked-up copy of your manuscript that highlights changes made to the original version. You should upload this as a separate file labeled 'Revised Manuscript with Track Changes'.
- An unmarked version of your revised paper without tracked changes. You should upload this as a separate file labeled 'Manuscript'.

If you would like to make changes to your financial disclosure, please include your updated statement in your cover letter. Guidelines for resubmitting your figure files are available below the reviewer comments at the end of this letter.

If applicable, we recommend that you deposit your laboratory protocols in protocols.io to enhance the reproducibility of your results. Protocols.io assigns your protocol its own identifier (DOI) so that it can be cited independently in the future. For instructions see: https://journals.plos.org/plosone/s/submission-guidelines#loc-laboratory-protocols. Additionally, PLOS ONE offers an option for publishing peer-reviewed Lab Protocol articles, which describe protocols hosted on protocols.io. Read more information on sharing protocols at https://plos.org/protocols?utm_medium=editorial-email&utm_source=authorletters&utm_campaign=protocols.

As the corresponding author, your ORCID iD is verified in the submission system and will appear in the published article. PLOS supports the use of ORCID, and we encourage all coauthors to register for an ORCID iD and use it as well. Please encourage your coauthors to verify their ORCID iD within the submission system before final acceptance, as unverified ORCID iDs will not appear in the published article. *Only*  the individual author can complete the verification step; PLOS staff *cannot*  verify ORCID iDs on behalf of authors.

We look forward to receiving your revised manuscript.

Kind regards,

Muhammad Shakaib, PhD

Academic Editor

PLOS One

Journal Requirements:

When submitting your revision, we need you to address these additional requirements.

1.Please ensure that your manuscript meets PLOS ONE's style requirements, including those for file naming. The PLOS ONE style templates can be found at

https://journals.plos.org/plosone/s/file?id=wjVg/PLOSOne_formatting_sample_main_body.pdf and

https://journals.plos.org/plosone/s/file?id=ba62/PLOSOne_formatting_sample_title_authors_affiliations.pdf

2. The ethical approval number(s) listed in the manuscript and/or submission metadata does not match the approval number on the ethical approval document you provided. Please ensure that all approval numbers are correct.

3. Thank you for stating the following financial disclosure:

“This study was supported by the Taipei City Hospital, Taiwan (TPCH-114-06).”

Please state what role the funders took in the study.  If the funders had no role, please state: "The funders had no role in study design, data collection and analysis, decision to publish, or preparation of the manuscript."

If this statement is not correct you must amend it as needed.

Please include this amended Role of Funder statement in your cover letter; we will change the online submission form on your behalf.

4. Please note that funding information should not appear in any section or other areas of your manuscript. We will only publish funding information present in the Funding Statement section of the online submission form. Please remove any funding-related text from the manuscript.

5. We note that you have indicated that there are restrictions to data sharing for this study. PLOS only allows data to be available upon request if there are legal or ethical restrictions on sharing data publicly. For more information on unacceptable data access restrictions, please see http://journals.plos.org/plosone/s/data-availability#loc-unacceptable-data-access-restrictions.

Before we proceed with your manuscript, please address the following prompts:

a) If there are ethical or legal restrictions on sharing a de-identified data set, please explain them in detail (e.g., data contain potentially identifying or sensitive patient information, data are owned by a third-party organization, etc.) and who has imposed them (e.g., a Research Ethics Committee or Institutional Review Board, etc.). Please also provide contact information for a data access committee, ethics committee, or other institutional body to which data requests may be sent.

b) If there are no restrictions, please upload the minimal anonymized data set necessary to replicate your study findings to a stable, public repository and provide us with the relevant URLs, DOIs, or accession numbers. For a list of recommended repositories, please see

https://journals.plos.org/plosone/s/recommended-repositories. You also have the option of uploading the data as Supporting Information files, but we would recommend depositing data directly to a data repository if possible.

We will update your Data Availability statement on your behalf to reflect the information you provide.

6. Your ethics statement should only appear in the Methods section of your manuscript. If your ethics statement is written in any section besides the Methods, please move it to the Methods section and delete it from any other section. Please ensure that your ethics statement is included in your manuscript, as the ethics statement entered into the online submission form will not be published alongside your manuscript.

7. We notice that your supplementary figures are uploaded with the file type 'Figure'. Please amend the file type to 'Supporting Information'. Please ensure that each Supporting Information file has a legend listed in the manuscript after the references list.

8. If the reviewer comments include a recommendation to cite specific previously published works, please review and evaluate these publications to determine whether they are relevant and should be cited. There is no requirement to cite these works unless the editor has indicated otherwise.

Additional Editor Comments:

The authors are suggested to consider all the reviewers’ comments, in particular improve the methodology in order to support their findings and conclusions.

[Note: HTML markup is below. Please do not edit.]

Reviewer's Responses to Questions

**Comments to the Author**

1. Is the manuscript technically sound, and do the data support the conclusions?

Reviewer #1: No

Reviewer #2: Partly

**********

2. Has the statistical analysis been performed appropriately and rigorously?

Reviewer #1: No

Reviewer #2: No

**********

3. Have the authors made all data underlying the findings in their manuscript fully available?

The PLOS Data policy

Reviewer #1: No

Reviewer #2: No

**********

4. Is the manuscript presented in an intelligible fashion and written in standard English?

Reviewer #1: Yes

Reviewer #2: Yes

**********

Reviewer #1: 1.The comparison between the SPMSQ and AD8 periods is difficult to interpret. The two periods differ not only in calendar time but also in cognitive screening instrument, follow-up duration, and population composition. Therefore, the apparent reversal of the association among never-married participants may reflect measurement heterogeneity rather than a true temporal change in the marital status–cognition relationship.

2.The AD8 assessment may introduce differential reporting bias by marital status. Because AD8 can be completed by either an informant or the participant, married individuals may be more likely to have spouse-based observations, whereas never-married individuals may rely more often on self-report or less frequent observers. This could lead to differential detection of cognitive decline and may partly explain the apparently protective association of never-married status in the AD8 period.

3.SPMSQ and AD8 are screening tools rather than clinical diagnostic instruments for MCI. The term “incident MCI” may overstate diagnostic certainty. The authors should consider using “screen-positive cognitive impairment” or “probable cognitive impairment” unless clinical diagnostic confirmation is available.

4.Marital status was treated as a fixed baseline exposure. Changes in marital status during follow-up, including widowhood, remarriage, separation, or changes in living arrangements, were not accounted for. This may introduce exposure misclassification, particularly in an older cohort.

5.The study lacks data on marital quality, loneliness, social network size, frequency of social participation, caregiver availability, technology use, and cognitive activities. These omissions limit mechanistic interpretation and raise the possibility of residual confounding.

6.The authors should report whether proportional hazards assumptions were tested, how competing risk of death was handled, how missing data were addressed, and whether multiple testing was considered in subgroup and interaction analyses.

7.The discussion is overly speculative.

Reviewer #2: While the study utilizes an impressive longitudinal cohort (n=103,863) and addresses a critical gap in measurement heterogeneity, several methodological concerns must be addressed to ensure the robustness of your findings.

Below are the formal recommendations for revision:

1. Introduction Section

• The Introduction should explicitly address the potential for reverse causality, a well-known challenge in social epidemiology. It is plausible that incipient cognitive decline, even in subclinical stages, may influence an individual's marital status or their likelihood of maintaining a long-term marriage. Highlighting this conceptual limitation early in the paper is essential for framing the study's theoretical boundaries before presenting the methodology.

2. Materials and Methods

• The cohort is derived from adults participating in voluntary government-sponsored health examinations. The authors should explicitly address the potential for selection bias, as this population is likely more health-conscious and possesses higher health literacy than the general elderly population in Taiwan.

• The patient selection process, as detailed in Figure 1 and the Methods section, reveals a significant loss of data that may compromise the representativeness of the findings. Specifically, the study excluded 44,254 individuals from the SPMSQ period and 32,700 from the AD8 period because they had a "single examination". This equates to approximately 42% of the initial cohort. In geriatric epidemiology, it is well-documented that participants who fail to return for follow-up assessments are typically frailer, more ill, or face more precarious socioeconomic conditions than those who remain in the study. By excluding nearly half of the original population, the results are highly susceptible to a "healthy volunteer" or "healthy survivor" bias, which may lead to an underestimation of the true associations between marital status and cognitive decline. To address this limitation, the authors should provide a comparative analysis of baseline characteristics (e.g., age, comorbidities, education, and marital status) between the included analytic sample and the individuals excluded due to having only a single examination. This comparison, which could be included in the supplementary material, is essential to evaluate the direction and magnitude of the potential selection bias.

• The study treats marital status as a "fixed characteristic" based on baseline data. Given that the mean follow-up duration reached 3.7 years for the SPMSQ period and 2.8 years for the AD8 period, it is highly probable that some participants experienced marital transitions (e.g., becoming widowed or divorced) during the course of the study. While other health variables were time-updated, keeping marital status fixed may introduce misclassification bias. I recommend treating marital status as a time-dependent covariate to align with the rest of the model.

• While the study aims to examine "incident mild cognitive impairment (MCI)," it is important to address a critical terminological distinction regarding the instruments employed. Neither the SPMSQ nor the AD8 questionnaire constitutes a formal clinical diagnosis of MCI. By design, both are screening instruments rather than diagnostic tools. I recommend that the authors avoid the definitive term "incident MCI" throughout the text. Instead, more methodologically accurate expressions such as "screen-positive cognitive impairment" or "probable cognitive impairment" should be used to reflect the limitations of using screening thresholds (SPMSQ ≥3 and AD8 ≥2) to define the study outcomes.

• There is a significant discrepancy between the "Covariates" section of the manuscript and the variables listed in the final analytical models (Tables 3–5). In the primary description of health-related covariates, the authors mention hypertension, diabetes, hyperlipidemia, heart disease, psychotropic or insomnia medication use, smoking, and alcohol. However, the notes for Table 3, Table 4, and Table 5, as well as a later subsection of the statistical analysis, include additional variables such as stroke, psychiatric history, and psychological distress. None of these additional variables are formally defined. The manuscript does not specify whether they were assessed via self-report, clinical diagnosis, or ICD codes from the database. The "Covariates" section should be updated to include an exhaustive and precise list of every variable included in the final models. Furthermore, the authors must explicitly define how stroke and psychiatric history were measured to allow for proper replication and validation of the results.

• While the models adjusted for "psychotropic or insomnia medication use" and "psychological distress", clinical depression is a potent confounder for both social isolation and cognitive test performance (particularly on the SPMSQ). If available, diagnostic data for depression should be included to minimize residual confounding.

4. Results

• The nature of the AD8 represents a major threat to the validity of the results for the "never-married" group. The AD8 relies on an informant to detect functional change. However, the data show that never-married individuals are far more likely to live alone (20%) compared to married individuals (0.9%). If a never-married participant lives alone and lacks a daily informant, the study likely relied on self-reporting, which is known to underestimate cognitive decline in early stages. The "protective" effect observed for never-married individuals in the AD8 period may simply be a misclassification error due to a lack of external observation.

• The mean follow-up for the SPMSQ period was 3.7 years, whereas the AD8 period was only 2.8 years. Cognitive decline is time-dependent. A shorter follow-up in the second period might only capture rapid-onset cases, while the first period captures a slower progression. While Cox models adjust for time-at-risk, this nearly one-year difference in observation density adds significant noise to the comparison of HRs between the two historical periods.

• The Results section frequently conflates objective data description with interpretation and discussion. In several passages, the authors do not merely report the findings but offer premature interpretations. For instance, the text states that “risk patterns reversed” and explicitly claims that “effect modification by sex and age was observed”. Such interpretive language and the labeling of statistical trends should be reserved for the Discussion section. The Results section should maintain a neutral tone, focusing strictly on the reporting of data, including Hazard Ratios, 95% Confidence Intervals, and interaction p-values, without assigning clinical or theoretical meaning at this stage.

5. Discussion

• The discussion attributes the risk reversal to "societal transformations" and the "digital revolution" between the 2005–2011 and 2012–2016 periods. However, this shift coincides exactly with the transition from the SPMSQ to the AD8. It is methodologically risky to suggest that social norms changed so radically in a single year (the transition point). The authors should prioritize the measurement artifact explanation over the social change hypothesis. It must be stated that the "temporal authenticity" claimed is highly questionable given the lack of an overlap period where both instruments were used simultaneously to calibrate results.

• The authors suggest that never-married individuals might possess "subtle cognitive advantages" or "greater adaptive capacity" in the second period. They overlook the fact that the AD8 is an informant-based instrument. The data show that never-married individuals are significantly more likely to live alone compared to married individuals. In the absence of a co-residing spouse to observe daily functional decline, the AD8 likely relied on self-reporting, which is prone to anosognosia (lack of insight) in early stages of impairment. The discussion must explicitly recognize that the "reduced risk" in this group is likely a detection bias resulting from the lack of a reliable informant.

**********

what does this mean?). If published, this will include your full peer review and any attached files.

If you choose “no”, your identity will remain anonymous but your review may still be made public.

**Do you want your identity to be public for this peer review?**  For information about this choice, including consent withdrawal, please see our Privacy Policy

Reviewer #1: No

Reviewer #2: No

**********

[NOTE: If reviewer comments were submitted as an attachment file, they will be attached to this email and accessible via the submission site. Please log into your account, locate the manuscript record, and check for the action link "View Attachments". If this link does not appear, there are no attachment files.]

To ensure your figures meet our technical requirements, please review our figure guidelines: https://journals.plos.org/plosone/s/figures

You may also use PLOS’s free figure tool, NAAS, to help you prepare publication quality figures: https://journals.plos.org/plosone/s/figures#loc-tools-for-figure-preparation.

NAAS will assess whether your figures meet our technical requirements by comparing each figure against our figure specifications.

# Author comment: Author response to Decision Letter 1

**DOI:** 10.1371/journal.pone.0359778.r002

26 Jun 2026

Please see our detailed, point-by-point response to the Editor's and Reviewers' comments in the attached "Point-by-Point Response" document, which addresses each comment individually with corresponding revisions, page numbers, and line numbers in the revised manuscript.

Attachment Submitted filename: Point-by-Point-Responses.docx

# Peer review: Decision Letter 1

**DOI:** 10.1371/journal.pone.0359778.r003

**Authors:** Muhammad Shakaib

5 Sep 2026

Dear Dr. Zhou,

Thank you for submitting your manuscript to PLOS One. After careful consideration, we feel that it has merit but does not fully meet PLOS One’s publication criteria as it currently stands. Therefore, we invite you to submit a revised version of the manuscript that addresses the points raised during the review process.

The authors are suggested to improve and extend the conclusions sections by including key findings, significance and contribution.

Please submit your revised manuscript by Oct 20 2026 11:59PM. If you will need more time than this to complete your revisions, please reply to this message or contact the journal office at plosone@plos.org. When you're ready to submit your revision, log on to https://www.editorialmanager.com/pone/ and select the 'Submissions Needing Revision' folder to locate your manuscript file.

- A letter that responds to each point raised by the academic editor and reviewer(s). You should upload this letter as a separate file labeled 'Response to Reviewers'.
- A marked-up copy of your manuscript that highlights changes made to the original version. You should upload this as a separate file labeled 'Revised Manuscript with Track Changes'.
- An unmarked version of your revised paper without tracked changes. You should upload this as a separate file labeled 'Manuscript'.

If you would like to make changes to your financial disclosure, please include your updated statement in your cover letter. Guidelines for resubmitting your figure files are available below the reviewer comments at the end of this letter.

If applicable, we recommend that you deposit your laboratory protocols in protocols.io to enhance the reproducibility of your results. Protocols.io assigns your protocol its own identifier (DOI) so that it can be cited independently in the future. For instructions see: https://journals.plos.org/plosone/s/submission-guidelines#loc-laboratory-protocols. Additionally, PLOS One offers an option for publishing peer-reviewed Lab Protocol articles, which describe protocols hosted on protocols.io. Read more information on sharing protocols at https://plos.org/protocols?utm_medium=editorial-email&utm_source=authorletters&utm_campaign=protocols.

As the corresponding author, your ORCID iD is verified in the submission system and will appear in the published article. PLOS supports the use of ORCID, and we encourage all coauthors to register for an ORCID iD and use it as well. Please encourage your coauthors to verify their ORCID iD within the submission system before final acceptance, as unverified ORCID iDs will not appear in the published article. *Only*  the individual author can complete the verification step; PLOS staff *cannot*  verify ORCID iDs on behalf of authors.

We look forward to receiving your revised manuscript.

Kind regards,

Muhammad Shakaib, PhD

Academic Editor

PLOS One

Journal Requirements:

If the reviewer comments include a recommendation to cite specific previously published works, please review and evaluate these publications to determine whether they are relevant and should be cited. There is no requirement to cite these works unless the editor has indicated otherwise.

Please review your reference list to ensure that it is complete and correct. If you have cited papers that have been retracted, please include the rationale for doing so in the manuscript text, or remove these references and replace them with relevant current references. Any changes to the reference list should be mentioned in the rebuttal letter that accompanies your revised manuscript. If you need to cite a retracted article, indicate the article’s retracted status in the References list and also include a citation and full reference for the retraction notice.

[Note: HTML markup is below. Please do not edit.]

Reviewers' comments:

Reviewer's Responses to Questions

**Comments to the Author**

Reviewer #2: All comments have been addressed

**********

2. Is the manuscript technically sound, and do the data support the conclusions?

Reviewer #2: (No Response)

**********

3. Has the statistical analysis been performed appropriately and rigorously?

Reviewer #2: (No Response)

**********

4. Have the authors made all data underlying the findings in their manuscript fully available?

The PLOS Data policy

Reviewer #2: (No Response)

**********

5. Is the manuscript presented in an intelligible fashion and written in standard English?

Reviewer #2: (No Response)

**********

Reviewer #2: (No Response)

**********

what does this mean?). If published, this will include your full peer review and any attached files.

If you choose “no”, your identity will remain anonymous but your review may still be made public.

**Do you want your identity to be public for this peer review?**  For information about this choice, including consent withdrawal, please see our Privacy Policy

Reviewer #2: No

**********

[NOTE: If reviewer comments were submitted as an attachment file, they will be attached to this email and accessible via the submission site. Please log into your account, locate the manuscript record, and check for the action link "View Attachments". If this link does not appear, there are no attachment files.]

To ensure your figures meet our technical requirements, please review our figure guidelines: https://journals.plos.org/plosone/s/figures

You may also use PLOS’s free figure tool, NAAS, to help you prepare publication quality figures: https://journals.plos.org/plosone/s/figures#loc-tools-for-figure-preparation.

NAAS will assess whether your figures meet our technical requirements by comparing each figure against our figure specifications.

# Author comment: Author response to Decision Letter 2

**DOI:** 10.1371/journal.pone.0359778.r004

12 Sep 2026

We sincerely thank the reviewer for the careful evaluation of our manuscript and are grateful for the positive assessment. We appreciate the reviewer's constructive comments throughout the review process, which have substantially improved the quality and clarity of our manuscript.

Attachment Submitted filename: Point-by-Point-Responses_2026.9.11.docx

# Editor report: Decision Letter 2

**DOI:** 10.1371/journal.pone.0359778.r005

**Authors:** Muhammad Shakaib

17 Sep 2026

Marital status and incident screen-positive cognitive impairment in a Taiwanese longitudinal cohort (2005–2016): Associations vary by cognitive screening instrument

PONE-D-26-10868R2

Dear Dr. Zhou,

We’re pleased to inform you that your manuscript has been judged scientifically suitable for publication and will be formally accepted for publication once it meets all outstanding technical requirements.

Within one week, you’ll receive an e-mail detailing the required amendments. When these have been addressed, you’ll receive a formal acceptance letter and your manuscript will be scheduled for publication.

An invoice will be generated when your article is formally accepted. Please note, if your institution has a publishing partnership with PLOS and your article meets the relevant criteria, all or part of your publication costs will be covered. Please make sure your user information is up-to-date by logging into Editorial Manager at Editorial Manager® and clicking the ‘Update My Information' link at the top of the page. For questions related to billing, please contact billing support.

If your institution or institutions have a press office, please notify them about your upcoming paper to help maximize its impact. If they’ll be preparing press materials, please inform our press team as soon as possible -- no later than 48 hours after receiving the formal acceptance. Your manuscript will remain under strict press embargo until 2 pm Eastern Time on the date of publication. For more information, please contact onepress@plos.org.

Kind regards,

Muhammad Shakaib, PhD

Academic Editor

PLOS One

Additional Editor Comments (optional):

Reviewers' comments:

# Editor report: Acceptance letter

**DOI:** 10.1371/journal.pone.0359778.r006

**Authors:** Muhammad Shakaib

PONE-D-26-10868R2

PLOS One

Dear Dr. Zhou,

I'm pleased to inform you that your manuscript has been deemed suitable for publication in PLOS One. Congratulations! Your manuscript is now being handed over to our production team.

At this stage, our production department will prepare your paper for publication. This includes ensuring the following:

* All references, tables, and figures are properly cited

* All relevant supporting information is included in the manuscript submission,

* There are no issues that prevent the paper from being properly typeset

You will receive further instructions from the production team, including instructions on how to review your proof when it is ready. Please keep in mind that we are working through a large volume of accepted articles, so please give us a few days to review your paper and let you know the next and final steps.

Lastly, if your institution or institutions have a press office, please let them know about your upcoming paper now to help maximize its impact. If they'll be preparing press materials, please inform our press team within the next 48 hours. Your manuscript will remain under strict press embargo until 2 pm Eastern Time on the date of publication. For more information, please contact onepress@plos.org.

You will receive an invoice from PLOS for your publication fee after your manuscript has reached the completed accept phase. If you receive an email requesting payment before acceptance or for any other service, this may be a phishing scheme. Learn how to identify phishing emails and protect your accounts at https://explore.plos.org/phishing.

If we can help with anything else, please email us at customercare@plos.org.

Thank you for submitting your work to PLOS One and supporting open access.

Kind regards,

PLOS One Editorial Office Staff

on behalf of

Dr. Muhammad Shakaib

Academic Editor

PLOS One
